# Rapid identification of *Acinetobacter baumannii* using novel specific molecular targets derived from pan-genome analysis and its clinical application

**DOI:** 10.3389/fmicb.2025.1669811

**Published:** 2025-10-29

**Authors:** Shuang Chen, Huayang Wang, Chen Ju, Pengliang Zhang, YongQiang Ren, Yaxuan Chen, Xiaoqi Yi, JianYing Zhang, Shenghang Zhang, Xinran Xiang

**Affiliations:** ^1^The First Affiliated Hospital of Henan University of Science and Technology, Luoyang, China; ^2^School of Life Science, Huaiyin Normal University, Huai’an, China; ^3^Fujian Key Laboratory of Aptamers Technology, Fuzhou General Actual Medical School (The 900th Hospital), Fujian Medical University, Fuzhou, China

**Keywords:** *Acinetobacter baumannii*, novel specific molecular targets, pan-genome analysis, PCR, nucleic acid testing

## Abstract

*Acinetobacter baumannii* is a pathogen capable of causing severe hospital-acquired infections such as ventilator-associated pneumonia and bacteremia, accounting for over 80% of nosocomial infections. Current nucleic acid tests (NATs) for *A. baumannii* suffer from limitations in specificity and sensitivity due to reliance on suboptimal targets. Therefore, this study aimed to identify novel, highly specific molecular targets for *A. baumannii* NATs using pan-genome analysis. A total of 9 specific molecular targets for *A. baumannii* were screened from 642 genome sequences by pan-genome analysis: *outO, ureE, rplY, bioF, menH_3, hemW, paaF_1, smpB and ppaX*. These specific species targets have been verified by BLAST and PCR of non-target strains to have 100% specificity for *A. baumannii*. The specificity of the 9 target genes was verified by PCR, and 3 pairs of different PCR primers were designed for each target gene to determine the best sensitivity of PCR method for each target. Corresponding qPCR detection methods of 9 targets was also established and that of *ureE* was screened with the lowest detection limit of 10^−7^ ng/μL. The qPCR method based on the *ureE* gene can achieve rapid, sensitive and accurate detection of *A. baumannii* in actual samples with interference from non-target bacteria. After verification of 23 samples, the qPCR method based on the mined target met the requirements in sensitivity, specificity and efficiency, and was consistent with the national verification method. ‌These results confirm that novel pan-genome targets with excellent generalizability among *A. baumannii* strains enhance detection accuracy in hospital environments, bringing hope for rapid clinical identification, timely interventions, and reduced mortality.

## 1 Introduction

*Acinetobacter baumannii* is a gram-negative, aerobic, non-fermenting opportunistic pathogen that has become a major threat for hospital infections (Yang et al., 2025), accounting for 20–30% of hospital-acquired infections in intensive care units globally (Murray et al., 2022). It is highly adapted to the environment, capable of surviving for extended periods, primarily spreading through contaminated medical equipment and direct contact, leading to nosocomial cross-infections. The most severe clinical manifestations include ventilator-associated pneumonia (VAP) and bloodstream infections (BSI), with mortality rates reaching as high as 52–66% and 34–43.4%, respectively (Yehya et al., 2025). In severe cases, it can lead to sepsis or multi-organ failure, significantly increasing the mortality rate of patients (Moazen et al., 2025). Rapid and accurate identification of the pathogen is therefore crucial for clinical diagnosis and infection control.

Phenotypic culture-based identification remains the gold standard for detecting *A. baumannii*, offering operational simplicity and inexpensive. However, this method requires 24–48 h to yield preliminary results (Purushothaman et al., 2024). In recent years, nucleic acid testing (NAT) such as PCR have become the predominant approach for *A. baumannii* identification due to their rapidity and efficiency, with the fundamental principle relying on specific recognition of conserved genomic sites (Abhari et al., 2021). The *bla_OXA-51-like_* carbapenemase gene cluster was proposed in 2005 as a species-specific molecular marker for *A. baumannii*, widely used for identification; however, studies have confirmed that this gene can be found in other *Acinetobacter* species through horizontal transfer, increasing the risk of misdiagnosis (Vijayakumar et al., 2019). More critically, the *bla_OXA-51-like_* gene cannot distinguish between highly pathogenic clinical strains and non-pathogenic variants found in the environment (Sotomayor et al., 2024). On the other hand, the *rpoB* gene shows pronounced conservation within the *Acinetobacter calcoaceticus-baumannii* complex, with a sequence similarity to *A. nosocomialis* exceeding 95% and to *A. pittii* exceeding 93%, making accurate species-level identification difficult (Sheck et al., 2023). The *gyrB* gene also shows deficiencies in species specificity and amplification efficiency, making it an unsuitable molecular identification target (Anggraini et al., 2022). Despite the superior speed and sensitivity of PCR compared to culture methods, its accuracy critically depends on species-specific conservation and discriminatory capacity of target genes. Consequently, identifying novel molecular targets that combine enhanced specificity with ultra-high sensitivity is paramount for advancing diagnostic precision in *A. baumannii* detection.

The scale of global microbial genome databases has expanded expeditiously with the proliferation of high-throughput sequencing technologies and concomitant reductions in sequencing costs. Notably, the National Center for Biotechnology Information (NCBI) database now hosts 40,338 *A. baumannii* genomes, representing a staggering increase from the mere 12 isolates available in 2010^1^. Concurrently, the European Nucleotide Archive (ENA) has seen its repository of *A. baumannii* sequences surge from 482 complete genomes in 2015 to 56,672 assembled genomes as of 2025^2^. This exponential growth in genomic resources provides a robust foundation for mining highly-specific diagnostic targets and advancing precision detection methodologies. Within this context, pan-genome analysis serves as a pivotal strategy for integrating genomic information across multiple strains, enabling systematic identification of conserved sequences shared among diverse pathogen isolates (Chaudhari et al., 2016). This approach effectively circumvents the limitations in specificity and sensitivity that arise from strain variability during conventional target selection (Chen et al., 2016). Compared with molecular targets derived from single reference genomes historically employed, the sequences identified through pan-genome analysis exhibit enhanced strain commonality and intra-species conservation, thereby significantly improving the accuracy and general applicability of detection methodologies. Several studies have used pan-genome analysis to determine new targets for pathogen detection, such as *Salmonella* (Ye et al., 2021) and *Listeria monocytogenes* (Li et al., 2021a), proving the feasibility of screening specific targets through pan-genome screening. To date, however, there have been no systematic reports on *A. baumannii*-specific, high-resolution molecular targets for nucleic acid diagnostics. The discovery of such targets would furnish precision molecular recognition elements for emerging platforms like CRISPR-based assays, thereby advancing pathogen diagnostic methodologies toward high-sensitivity and high-specificity paradigms.

**Figure 1:**
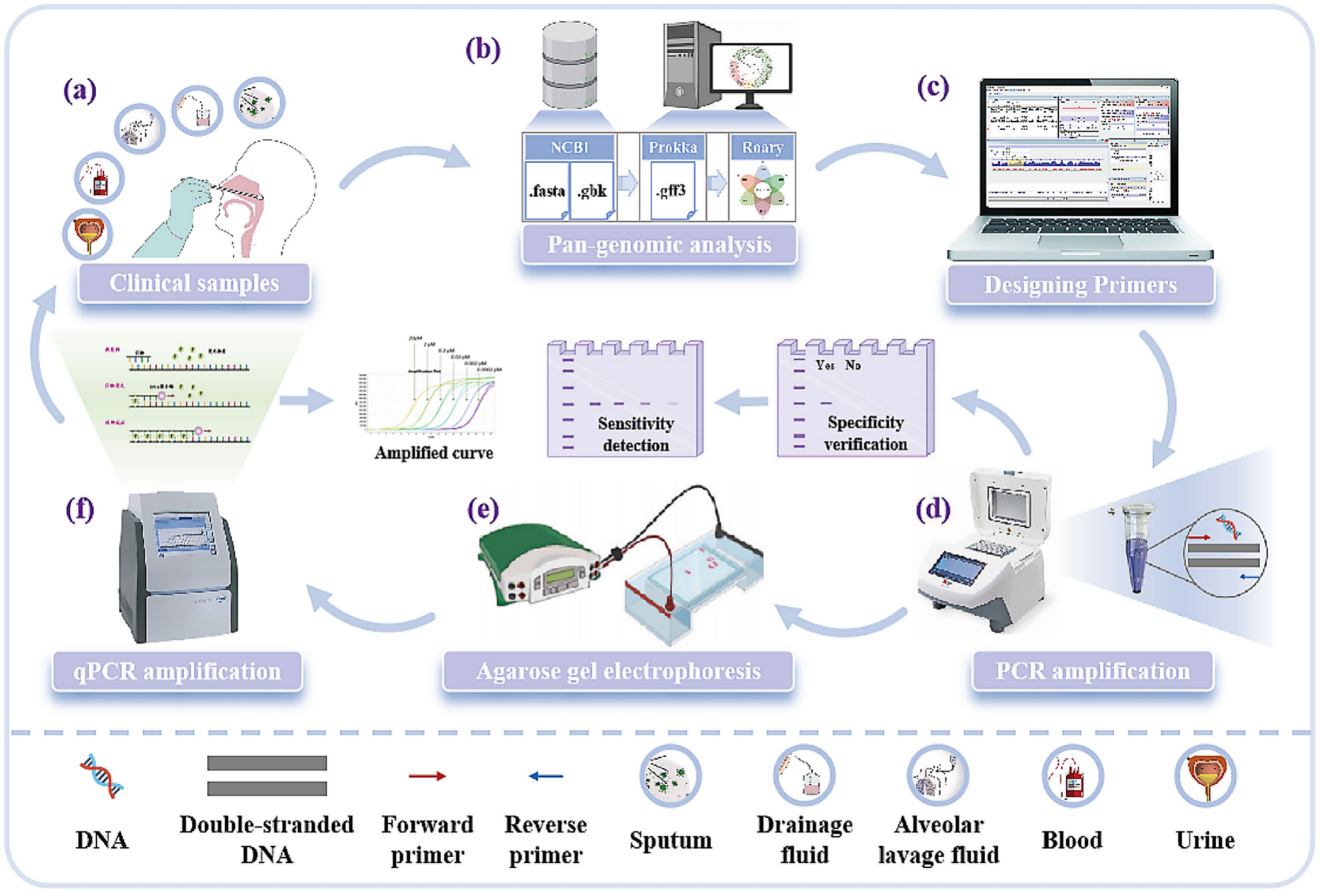
Experimental process for the discovery of new molecular targets of *A. baumannii*. **(a)** Five clinical samples of *A. baumannii*: Sputum, drainage fluid, alveolar lavage fluid, blood, and urine. **(b)** Mining new molecular targets of *A. baumannii* using pan-genomic analysis techniques. **(c)** Designing primers for molecular targets. **(d)** Validation of target specificity and sensitivity by PCR amplification. **(e)** Analysis of PCR amplification results based on fluorescent bands from agarose gel electrophoresis. **(f)** Validation of target sensitivity by qPCR and reading of standard curves.

In this study, the clinically-optimized *A. baumannii* pan-genomic target mining diagnostic system was developed. This system utilizes pan-genome analysis technology to comprehensively screen conserved genomic elements across *A. baumannii* populations. By analyzing core genomic regions while systematically excluding horizontally transferred mobile elements, it identifies novel molecular targets exhibiting minimal variation. Compared to conventional targets, these newly identified sequences demonstrate enhanced species specificity and cross-strain conservation, providing a robust molecular foundation for precision diagnostics. This study aims to discover novel molecular targets through pan-genome analysis, with subsequent verification of their diagnostic capabilities via PCR and qPCR assays using clinically representative isolates, ultimately investigating the applicability of these targets in *A. baumannii* detection for point-of-care deployment (Figure 1).

## 2 Materials and methods

### 2.1 Mining of new molecular targets of *A. baumannii* target genes

To identify *A. baumannii* species-specific targets, whole-genome sequences of 642 *A. baumannii* and 28 other non-*A. baumannii* strains were acquired from the National Center for Biotechnology Information (NCBI) genome database. Detailed information about the sequences is shown in Supplementary material 1. All analyzed genome sequences were re-annotated with the Prokka v1.14.6 software and the output was used to construct the whole-genome map by Roary v3.13.0 (Li et al., 2021b; Shang et al., 2020). The core genome was determined for each isolate using a 99% cutoff and a 85% BLASTP identity cutoff (Pang et al., 2019). Harvest v1.1 was used to align the core genome of *A. baumannii* (Treangen et al., 2014). The criteria for selecting species-specific genes of *A. baumannii* were the 100% presence of the genes in all target *A. baumannii* strains and their complete absence in all non-*A. baumannii* strains. The specific gene detected was compared with the nucleotide sequence of NCBI using BLAST to confirm its specificity further. Ultimately only nine genes (*outO*, *ureE*, *rplY*, *bioF*, *menH_3*, *hemW*, *paaF_1*, *smpB*, *ppaX*) met all the stringent criteria and were therefore selected for subsequent experimental validation.

### 2.2 Experimental strains and DNA extraction

A total of 152 *A. baumannii* strains isolated from clinical samples collected from patients of different ages and genders in hospitals and 27 non-*A. baumannii* strains used in this study are listed in Supplementary Table S1. All strains were inoculated into Columbia blood agar medium (Comark, China) for 18–24 h at 37 °C. Single strains obtained were subcultured in Luria-Bertani broth (Huankai Microbiology). Genomic DNA was extracted from the recultured strains using the bacterial DNA extraction kit (Tiangen Biochemical, China). The extracted strains were added with 30% glycerol and stored at −20 °C.

### 2.3 Procedures of PCR and qPCR

The 20 μL PCR reaction contained 10 μL of 2 × PCR Mix (Dongsheng Biotechnology, China), 0.8 μL of template DNA, 0.8 μL of each forward and reverse primer (10 μM), and 8.4 μL of ddH_2_O. Thus, the final concentration of each primer in the reaction system is 0.4 μM. A Bio-Rad Bole T100 gradient PCR instrument was used for PCR amplification, with the following cycling conditions: pre-denaturation at 94 °C for 3 min; followed by 35 cycles of denaturation at 94 °C for 30 s, 60 °C for 30 s and 72 °C for 30 s, and a final extension at 72 °C for 5 min. PCR amplification products were identified by a 1.7% agarose gel electrophoresis, which were visualized using an automatic digital gel image analysis system (Tanon-2500; Tanon Science & Technology Co., Ltd., Shanghai, China).

The 20 μL qPCR reaction mixture contained: 10 μL of 2 × Q3 SYBR PCR Master Mix, 0.4 μL of each forward and reverse primer (10 μM), 1 μL of genomic DNA, and 8.6 μL of ddH₂O. Thus, the final concentration of each primer in the reaction system is 0.2 μM. The qPCR cycling conditions were as follows: pre-denaturation at 95 °C for 30 s; followed by 40 cycles of denaturation at 95 °C for 10 s, and annealing/extension at 60 °C for 30 s. A standard curve was generated by plotting the logarithm of the template DNA concentration against the mean cycle threshold (Ct) value obtained for each dilution. The limit of detection (LOD) of each primer pair was defined as the lowest template concentration that yielded a consistent positive amplification signal (Ct ≤ 40, with a characteristic sigmoidal amplification curve and a distinct melting curve peak) in three independent replicates. Melting curve analysis was performed to evaluate positive or negative qPCR results: starting at 65 °C for 5 s, then ramping to 95 °C at a rate of 0.5 °C/s. Ct values for *A. baumannii* in the standard samples were recorded, and SYBR fluorescence quantitative PCR amplification curves were plotted. Samples were considered positive if the amplification curve was sigmoidal with Ct ≤ 35. Samples with Ct ≥ 40 were negative. For Ct values between 35 and 40, repeat testing was performed: results were deemed positive if the repeat Ct was < 40 with a distinct peak-shaped amplification curve; otherwise, they were negative.

PCR testing validates the specificity and feasibility of the newly designed primers, while qPCR is used for quantifying the sensitivity and detection limit of the target gene, particularly in the application of complex clinical samples. These two methods are carried out in sequence, with PCR serving as a preliminary screening tool, and qPCR providing the quantitative accuracy required for clinical diagnosis.

### 2.4 Design and validation of *A. baumannii* species-specific primers

In order to achieve rapid and efficient detection of *A. baumannii*, it is critical to select primers with high sensitivity and specificity. For each excavated species-specific targets of *A. baumannii*, 3 groups of PCR primers and 1 group of qPCR primers were, respectively, designed using Oligo7.0 software (Supplementary Table S2). The specificity of all primers was verified using DNA extracted from *A. baumannii* including standard strains and clinical isolates listed in Supplementary Table S2 with ultrapure water as the negative. To determine the applicability and accuracy of primers, the LOD of specific primers was evaluated in a PCR assay. To detect the sensitivity of the designed primers, continuous 10-fold gradient dilution of genomic DNA solution from standard strains (ranging from 10^−1^ to 10^−7^ ng/μL) was used for PCR/qPCR detection. According to the linear relationship between DNA concentration and pixel intensity (PCR)/Ct value, a standard curve was established to determine LOD, and the most sensitive primers were selected for subsequent detection of actual samples.

### 2.5 Interference experiment based on nontarget strains

To evaluate the stability and accuracy of the qPCR method for the novel specific targets, interference from actual samples and non-target strains was investigated following the method described previously (Zhou et al., 2017). Selected human sputum samples and non-target strains – such as *Klebsiella pneumoniae* and *Staphylococcus aureus*, which are common in clinical settings and may coexist with multidrug-resistant *A. baumannii* in the respiratory tract or other samples. *Klebsiella pneumoniae* and *Staphylococcus aureus* were used in interference experiments to simulate potential cross-reactivity in real molecular diagnostics. For spiking experiments, sputum samples were collected from healthy person, separated on a clean bench, and plate count was performed to confirm no interference from other bacteria before use. Then 1 g of prepared sputum samples were mixed with 9 mL of sterile normal saline to obtain a liquid sample matrix. Serial dilutions of a target bacterial suspension were added to sputum sample matrix to final concentrations ranging from 2.5 × 10^1^ to 10^7^ CFU/g. These samples were then spiked with a fixed concentration (10^6^ CFU/mL) of non-target bacteria (*Klebsiella pneumoniae* and *Staphylococcus aureus*), along with serial dilutions of *A. baumannii* ranging from 10^1^ to 10^7^ CFU/mL. This mixture is then used for DNA extraction and qPCR analysis. LODs were calculated using standard curves and the effects of spiked samples and non-target strains were analyzed. All experiments were performed in triplicate, and data are presented as mean ± standard deviation (SD). Statistical comparisons were performed using one-way ANOVA where appropriate, with a *p*-value < 0.05 considered statistically significant.

### 2.6 Detection of *A. baumannii* in actual sample

To verify the feasibility of detection of *A. baumannii* in actual samples, the 96 clinical samples (including 37 sputum samples, 28 alveolar lavage fluid samples, 13 drainage fluid samples, 9 blood samples, 9 Urine samples) were randomly selected from the hospital. These clinical samples were processed for DNA extraction. For samples with low bacterial load (e.g., blood and urine), a short-term enrichment culture was performed by inoculating the sample into Luria-Bertani broth and incubating at 37 °C for 4–12 h with shaking at 200 rpm. Genomic DNA was then extracted from all samples according to the manufacturer’s instructions. The extracted DNA was subsequently subjected to PCR and qPCR analysis. This study compared the results of the designed PCR and qPCR analysis with those of the conventional culture method to evaluate the detection status of the newly mined molecular targets of *A. baumannii* in the identification of clinical samples.

## 3 Results

### 3.1 Novel species-specific targets for *A. baumannii*

Through pan-genome analysis and BLAST, 15 candidate genes were initially identified from the genome sequences of *A. baumannii* retrieved from the NCBI database. However, 6 of these candidates were excluded from further validation via *in silico* analysis, mainly due to either insufficient conservation across all *A. baumannii* strains or potential cross-reactivity with non-*A. baumannii* species. Ultimately, 9 specific target genes (*outO*, *ureE*, *rplY*, *bioF*, *menH_3*, *hemW*, *paaF_1*, *smpB*, and *ppaX*) were selected, adhering to the following criteria: 100% presence in all tested *A. baumannii* strains and complete absence in non-*A. baumannii* strains. The sequence data of these 9 specific genes are presented in Supplementary Table S3, which further confirms the high specificity of the newly identified targets for *A. baumannii* identification. Among these targets, the *outO* gene is functionally associated with the synthesis of a type 4 prepilin-like protein leader peptide-processing enzyme (LaPointe and Taylor, 2000). Additionally, a comparison was conducted between the newly mined species-specific targets and previously reported ones (e.g., *virulence* genes; Bhagirath et al., 2022), revealing that the detection targets screened in this study are more comprehensive than those in previous reports. Collectively, these results indicate that the gene targets identified in this study are suitable for the detection of *A. baumannii.*

### 3.2 Feasibility of PCR primer sets

**Figure 2:**
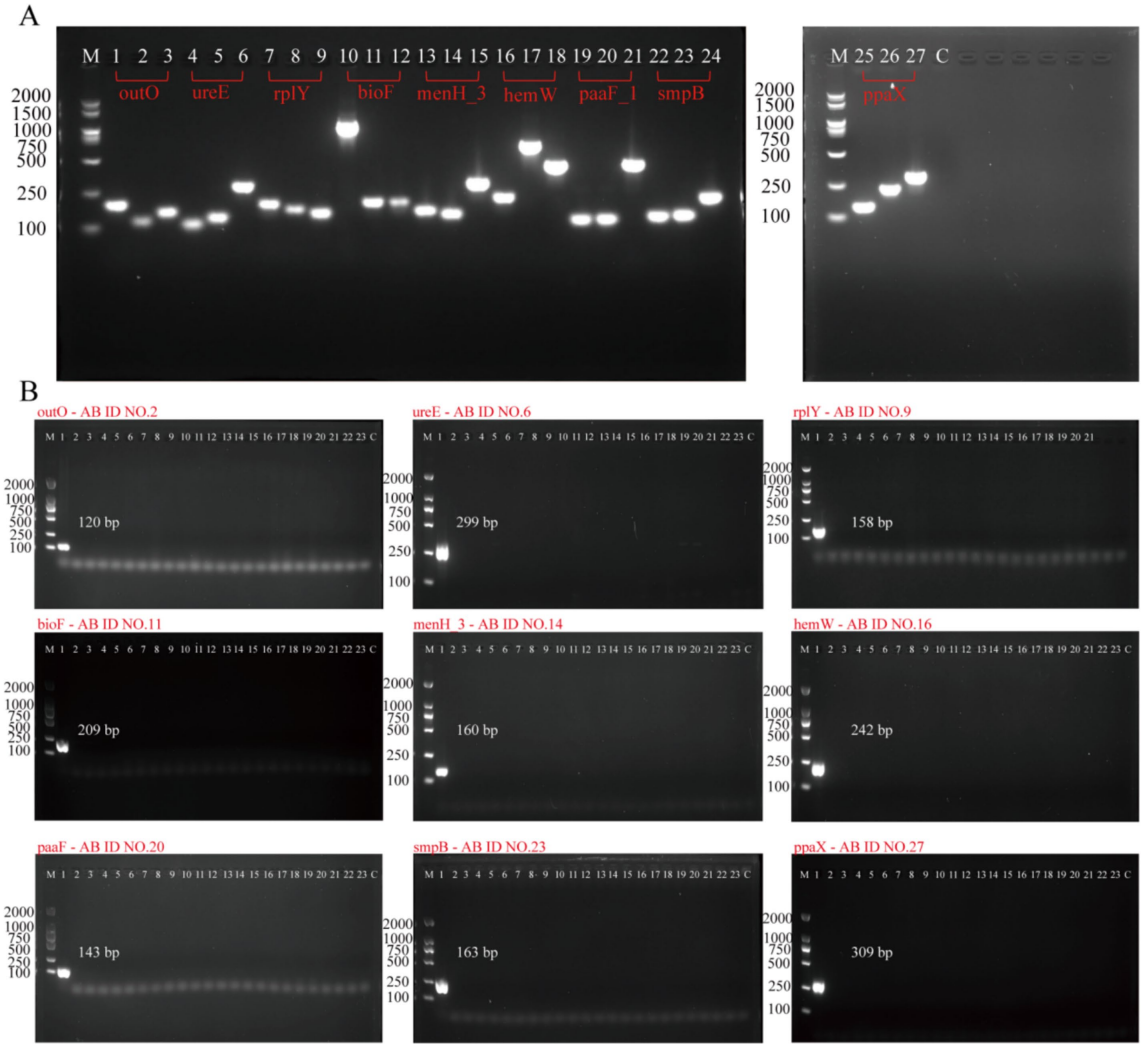
Evaluation of the PCR primers for *A. baumannii* molecular targets. **(A)** Feasibility analysis of all 27 primers: Agarose gel electrophoresis was performed to assess amplification efficacy. Lane M: 100–2000 bp DNA size marker. Lanes 1–27: Amplification products of 27 PCR primers, respectively. Lane C: Negative control. **(B)** Specificity validation of nine primers: Nine primers (AB ID NO.2, 6, 9, 11, 14, 16, 20, 23, 27) were selected based on initial feasibility via gel electrophoresis. Lane M: 100–2000 bp DNA size marker. Lanes 1–23: Amplification products of primers tested against *A. baumannii* and 22 non-*A. baumannii* strains. Lane C: Negative control.

According to the method in Section 2.3, 27 pairs of primers (AB ID NO.1–27) were designed for 9 targets for PCR verification (Supplementary Table S2). After the completion of the PCR reaction, the products were identified by 1.7% agarose gel electrophoresis. As shown in Figure 2A, agarose gelelectrophoresis results demonstrated that all 27 pairs of primers could amplify specific bands of the expected size (lanes 1–27), with no non-specific amplification or primer dimers present. The negative control (Lane C) showed no bands, indicating that the reaction system was free of contamination. This result confirms that all primers have good amplification efficiency and feasibility, laying the foundation for subsequent specificity and sensitivity validation.

The specificity of nine targets (*outO, ureE, rplY, bioF, menH_3, hemW, paaF_1, smpB, ppaX*) was validated using standard strain (*Acinetobacter calcoaceticus*) and 22 *non-A. baumannii* strains (Supplementary Table S1). To detect *A. baumannii* in positive samples, PCR was employed to amplify the target genes including *outO, ureE, rplY, bioF, menH_3, hemW, paaF_1, smpB, ppaX, 16S rDNA, blaoxa-23, blaoxa-51-like, recA, gyrB, and gyrA* sites. PCR amplification showed that: All new target primers (*outO, ureE, rplY, bioF, menH_3, hemW, paaF_1, smpB, ppaX*) amplified the target band only in *A. baumannii*, and there was no specific amplification for other pathogens (Figure 2B), which confirmed that these targets could effectively distinguish *A. baumannii* from other *Acinetobacter* species. Bioinformatics analysis further confirmed that these targets are effective in distinguishing *A. baumannii* from other *Acinetobacter* species. These results indicate that *outO*, *ureE*, *rplY*, *bioF*, *menH_3*, *hemW*, *paaF_1*, *smpB*, and *ppaX* are specific to *A. baumannii*, and they outperform the target genes previously reported for the detection of *A. baumannii* (detailed information can be found in Supplementary Table S4).

### 3.3 Species-specific primer optimization

**Figure 3:**
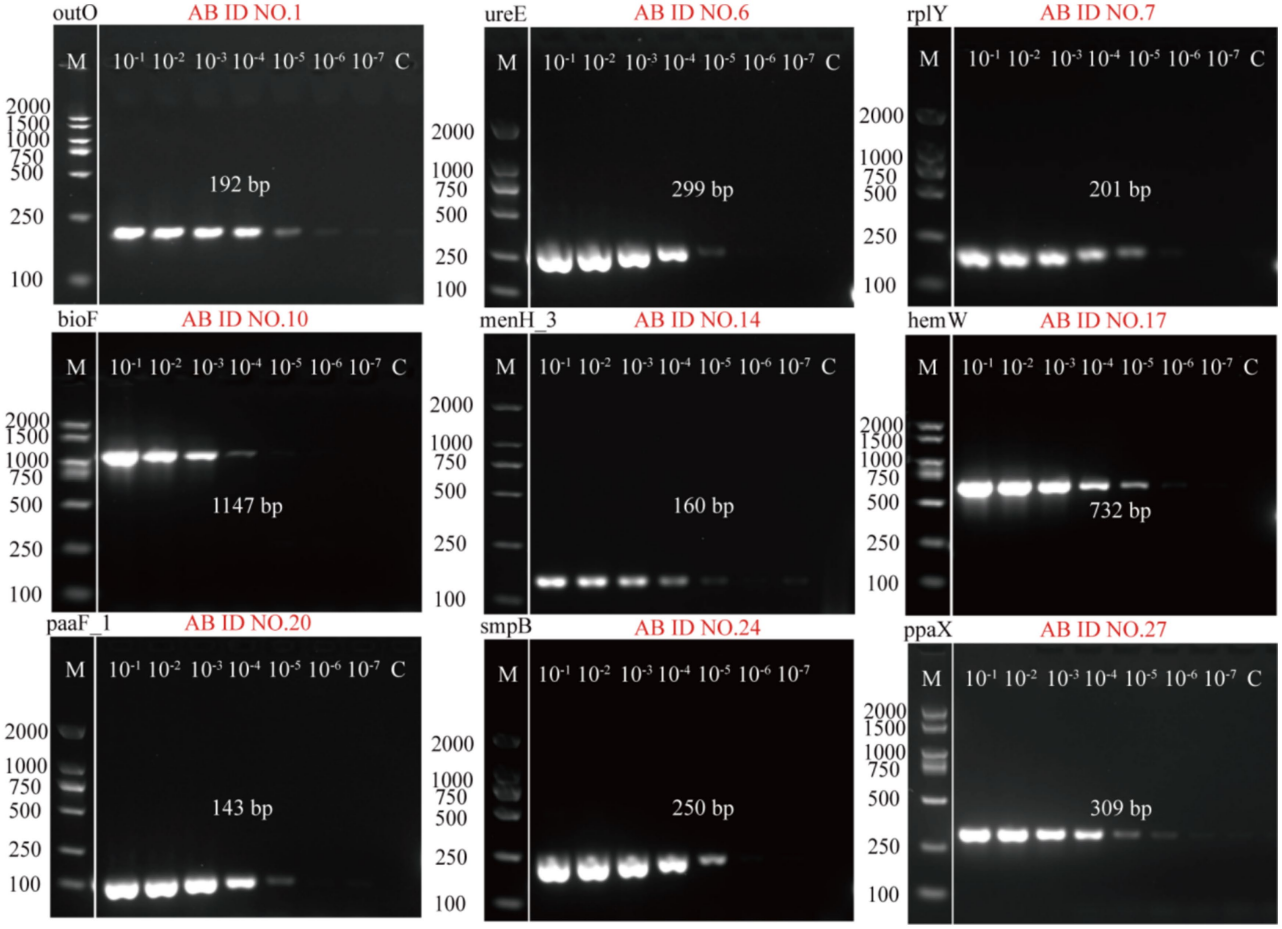
Agarose gel electrophoresis results of the sensitivity of 9 optimal primers detected by PCR method. AB ID NO.1, AB ID NO.6, AB ID NO.7, AB ID NO.10, AB ID NO.14, AB ID NO.17, AB ID NO.20, AB ID NO.24, AB ID NO.27 were screened from 27 primers designed based on the nine *A. baumannii* target genes.

The 10-fold diluted solutions of *A. baumannii* genomic DNA (ranging from 10^−1^ to 10^−7^ ng/μL) were used as the template DNA to prepare the PCR reaction system. The 27 PCR primers (AB ID NO.1-AB ID NO.27) were tested to select the optimal primer (one per target) based on sensitivity. Electrophoresis results of the 27 PCR primers were presented in Supplementary Figure S1. The standard curves of the PCR primers were made based on Image J analysis of fluorescence intensity, which were showed in Fig S2. After comparison, it was found that AB ID 1-F/R, AB ID 6-F/R, AB ID 7-F/R, AB ID 10-F/R, AB ID 14-F/R, AB ID 17-F/R, AB ID 20-F/R, AB ID 24-F/R, AB ID 27-F/R are more sensitive than other primers. Therefore, these primers were selected as optimization primers, where AB ID NO.1-F/R (*outO*) and AB ID NO.27-F/R (*ppaX*) exhibited higher sensitivity relative to other optimized primers (Figure 3).

### 3.4 Specificity and sensitivity of qPCR for the *A. baumannii* detection

**Figure 4:**
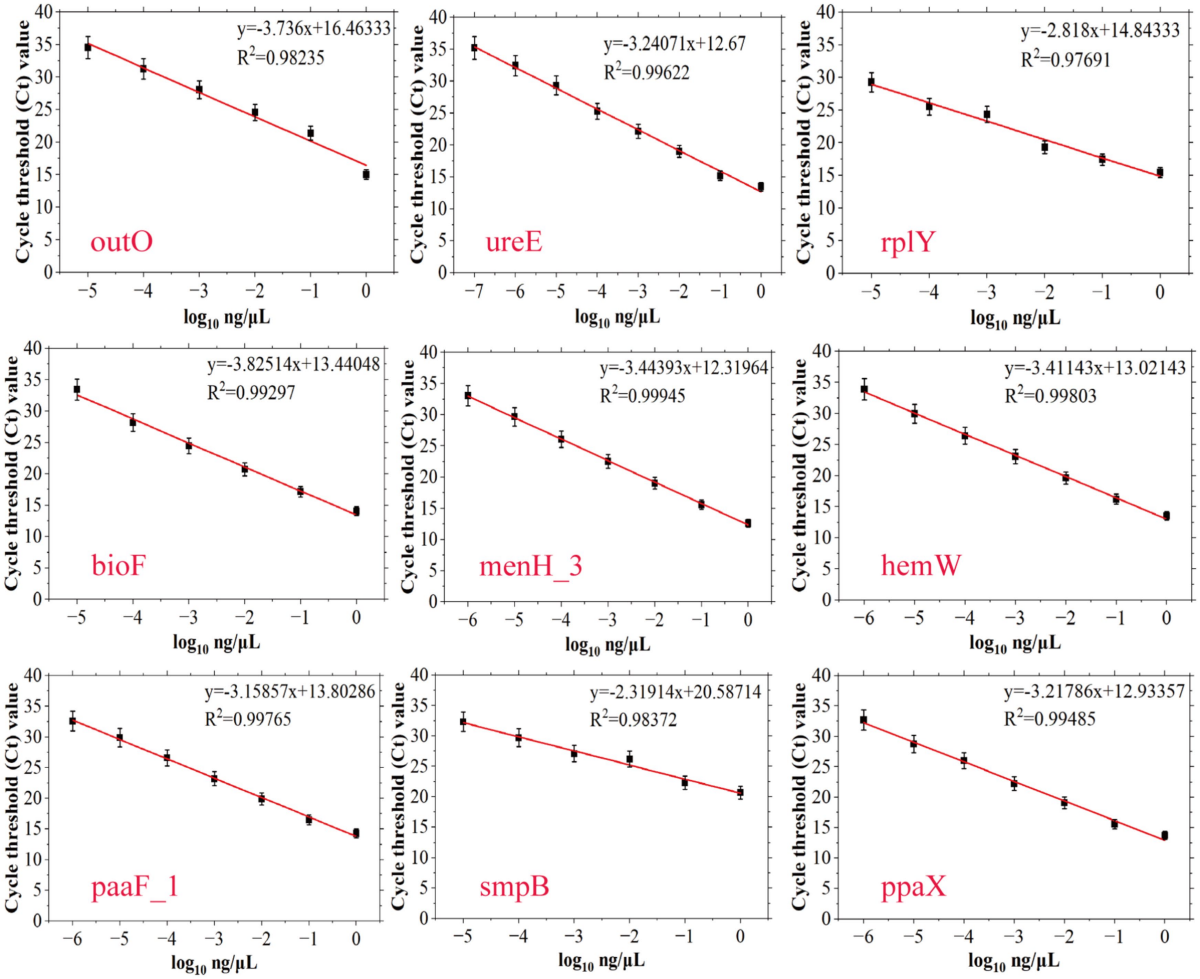
Establishment of standard curves of nine novel targets of *A. baumannii* (*outO*, *ureE*, *rplY*, *bioF*, *menH_3*, *hemW*, *paaF_1*, *smpB* and *ppaX*) by plotting cycle threshold (Ct) values against the log numbers of *A. baumannii* genomic DNA in a range of 10^0^–10^−7^ ng/μL. The error bars indicate the standard deviations derived from three measurements (*n* = 3).

In order to achieve accurate quantitative analysis, qPCR primers corresponding to 9 species-specific targets were designed (Supplementary Table S2, AB ID NO.28–AB ID NO.36). A standard strain of *A. baumannii* and 27 non-*A. baumannii* were used to verify the specificity of the qPCR primers, and the qPCR results were consistent with the PCR results as shown in Supplementary Table S5, demonstrating the high specificity of qPCR primers for *A. baumannii*. Standard curves were established based on these specie-specific targets to investigate the quantitative ability of different targets for *Acinetobacter baumannii* DNA. SYBR fluorescence quantitative amplification curves and melting curve were plotted in Supplementary Figure S3. In qPCR detection of *A. baumannii* genomic DNA, Ct values exhibited an inverse correlation with template concentration. Below the LOD of assay, Ct values either exceeded the instrument’s reliable quantification range or were statistically indistinguishable from negative controls. As shown in Figure 4, the four standard curves exhibited ideal linear correlations, with the *R*^2^ values ranged from 0.97691 to 0.99945. The LOD values of qPCR detection methods were, respectively, determined to be 10^−7^ ng/μL (approximately 15 copies/μL for *ureE*), 10^−6^ ng/μL (approximately 150 copies/μL for *menH_3*, *hemW*, *paaF_1*, *ppaX*), and 10^−5^ ng/μL (approximately 1,500 copies/μL for *outO*, *rplY*, *bioF*, *smpB*) by amplification curve analysis. All LOD values were derived from three independent replicates. To sum up, AB ID NO.29-F/R (*ureE*) has the best sensitivity and was used for subsequent spiked and actual sample detection.

Although all nine targets demonstrated high specificity and varying levels of sensitivity, the *ureE* gene (AB ID NO.29-F/R) exhibited the lowest detection limit (10^−7^ ng/μL) among the nine targets. Therefore, the *ureE*-based qPCR method was selected for subsequent spiking experiments and clinical sample detection due to its superior sensitivity and robustness, ensuring reliable performance even in complex sample matrices.

### 3.5 Detection of *A. baumannii* in sputum samples spiked with nontarget strains

**Figure 5:**
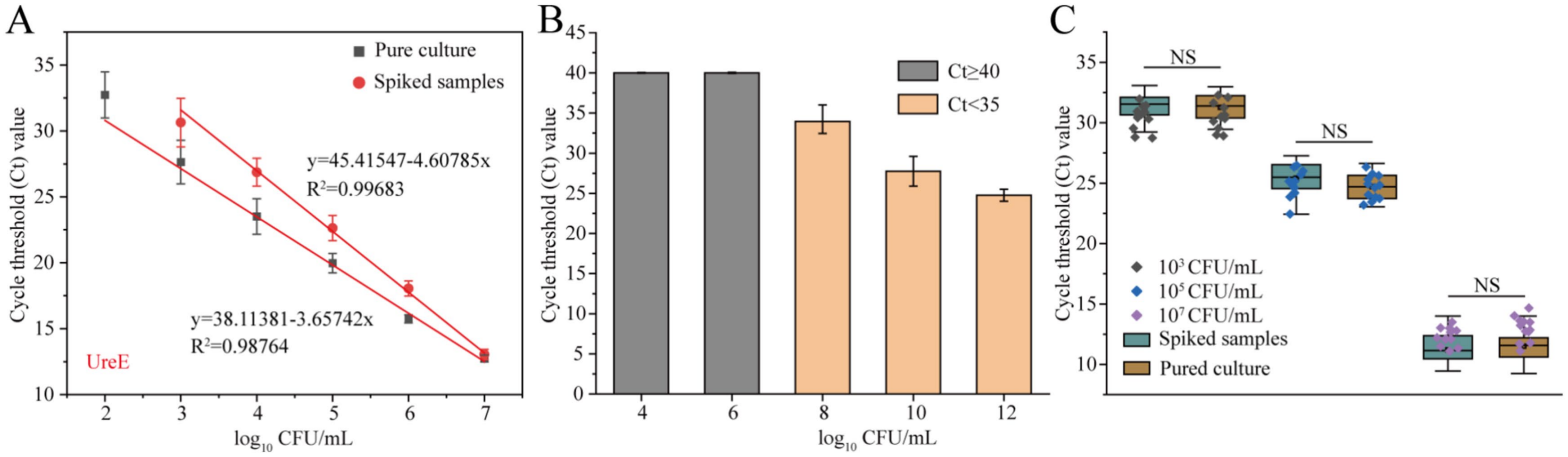
Evaluation of the practical performance of the *ureE* gene-based qPCR method. **(A)** Standard curves of pure culture and spiked samples with 10^6^ CFU/mL of non-target strains; **(B)** Comparison of Ct values of qPCR assays for detection of spiked samples with 10^1^ CFU/mL after different culture times (4–12 h); **(C)** Anti-interference of the *ureE* gene-based qPCR method by adding non-target strains (10^6^ CFU/mL) to high, medium and low concentration samples. Pure culture refers to the genomic DNA extracted from *A. baumannii* strains without any added matrix or non-target bacterial interference.

Serial 10-fold dilutions of pure-cultured strains of the *A. baumannii* were prepared and added to healthy human sputum samples spiked with 10^6^ CFU/mL of nontarget strains to achieve final concentrations of 10^1^–10^7^ CFU/mL. As shown in Figure 5A, the Ct values of the spiked samples at the low target *A. baumannii* concentration were slightly higher than those of the pure samples, but the standard curves of both maintained a good linear relationship, which indicated that the influence of the sample matrix on this method was limited. Although the samples with a spiking of less than 10^3^ CFU/mL could not be directly detected, the Ct value of the qPCR method in spiked samples with 10^1^ CFU/mL decreased to 27.74 ± 1.86 after 10 h of enrichment culture, which could be well judged as positive (Figure 5B). Furthermore, under the interference of non-target strains, the Ct values in sputum samples spiked with three different concentrations of target bacteria showed no significant differences from those in pure cultures (Figure 5C). These results suggest that this developed *ureE* gene-based qPCR assay with can detect *A. baumannii* without interference from stromal and non-target bacteria.

### 3.6 Detection of *A. baumannii* in actual clinical samples

To determine the feasibility of *A. baumannii* detection in actual samples based on nine novel target, 96 samples were tested using our proposed qPCR method (Table 1). For comparison, the protocol based on traditional biochemical culture was used to identify target *A. baumannii* strains in these clinical samples. The detection rates of the five clinical samples were 35.13% of sputum, 21.42% of alveolar lavage fluid, 23.07% of drainage fluid, 44.44% of blood and 11.11% of urine, respectively. Most positive samples can be detected without enrichment culture, with only one blood and urine sample requiring re-enrichment conditions to be detected. These results indicate that the qPCR method based on novel mined targets has a good consistency with the national the standard culture method (approximately 7 days) (Gupta et al., 2019) and has significant advantages in testing clinical samples with high and medium concentration of *A. baumannii*.

**Table 1:** Clinical sample detection form of *A. baumannii* targets.

Sample type	Sample size	Number of qPCR positive samples		Number of positive samples by culture method
		Without enrichment	With enrichment	
Sputum	37	11	13	13
Alveolar lavage fluid	28	4	6	6
Drainage fluid	13	2	3	3
Blood	9	2	3	4
Urine	9	0	1	1

## 4 Discussion

This research employed pan-genomic analysis to identify novel targets against *A. baumannii* and the specificity of these targets was successfully confirmed through genomic database alignment and PCR methods. Utilizing these targets for *A. baumannii* nucleic acid detection has the potential to overcome its limitations in specificity and sensitivity, thereby reducing the risk of serious hospital-acquired diseases like ventilator-associated pneumonia and bacteremia.

*Acinetobacter baumannii* is frequently encountered in healthcare settings and clinical specimens. Therefore, the detection of this bacterium, particularly multidrug-resistant strains, is of critical importance. Previous studies employed methods based on culture and biochemical reaction identification to classify *A. baumannii* species, typically using virulence genes specific to the target strains (Sheck et al., 2023). However, these approaches face significant limitations: (1) Variable expression levels of virulence genes due to strain heterogeneity or environmental factors may lead to false negatives (Sato et al., 2017); (2) Horizontally transferred genes like *bla_OXA-51-like_* exhibit cross-species homology, risking false positives (Vijayakumar et al., 2019; Martins et al., 2018); (3) Conventional PCR targets (e.g., *rpoB*, *gyrB*) lack sufficient discriminatory power within the *Acinetobacter calcoaceticus-baumannii* complex (Sheck et al., 2023; Anggraini et al., 2022). These deficiencies underscore the urgent need for highly specific molecular targets.

In this study, we leveraged pan-genome analysis of 642 genomic sequences across diverse *A. baumannii* strains to identify nine novel species-specific targets (*outO*, *ureE*, *rplY*, *bioF*, *menH_3*, *hemW*, *paaF_1*, *smpB*, *ppaX*). This strategy—validated in prior studies for *Salmonella* (Ye et al., 2021) and *Listeria monocytogenes* (Li et al., 2021a)—ensures comprehensive genomic coverage and minimizes target variability. Crucially, none of the nine target fragments were amplified in the 22 non-*A. baumannii* strains (Figure 2), outperforming traditional markers like *bla_OXA-51-like_*. This high specificity stems from our stringent selection criteria: targets were conserved across *A. baumannii* core genomes while absent in related species, effectively eliminating false positives from horizontal gene transfer events.

The qPCR assays exhibited exceptional sensitivity, with detection limits ranging from 1 × 10^−1^ to 1 × 10^−7^ ng/μL. Notably, the *ureE* target achieved the lowest LOD (1 × 10^−7^ ng/μL), surpassing the sensitivity of most reported targets. This sensitivity advantage was maintained in complex matrices: spiked sputum samples required only 10 h enrichment for detection at 2.5 × 10^6^ CFU/mL, suggesting clinical utility in direct specimen testing. Furthermore, our PCR/qPCR results showed perfect concordance with culture methods in 96 clinical samples (Table 1). This approach reduced the detection time from 7 days to 2 h—a critical advancement for ICU settings where rapid diagnosis impacts mortality (Murray et al., 2022). The specificity of the nine species-specific molecular targets was further confirmed by BLAST alignment and PCR verification.

In actual clinical, five types of clinical samples—sputum, alveolar lavage fluid, drainage fluid, blood, and urine—were evaluated for *A. baumannii* detection using the novel *ureE*-based qPCR assay. Among these, sputum and alveolar lavage fluid exhibited the highest positivity rates (35.13 and 21.42%, respectively), which is consistent with previous reports indicating that respiratory samples are the most common sources of *A. baumannii* isolation in ventilator-associated pneumonia (VAP) cases (Yang et al., 2025; Yehya et al., 2025). Blood samples showed a notably high detection rate (44.44%), underscoring the clinical severity of bacteremia caused by *A. baumannii*, though the sample size was limited. Urine samples had the lowest detection rate (11.11%), likely due to the lower bacterial load in urinary tract infections relative to respiratory or bloodstream infections. These findings align with existing literature suggesting that sample type significantly influences detection sensitivity, with respiratory and blood samples being more reliable for early diagnosis of *A. baumannii* infections (Murray et al., 2022; Sotomayor et al., 2024).

To the best of our knowledge, this is the first time these target genes have been used to detect *A. baumannii*. The functional roles of these targets are distinct from virulence-associated genes traditionally used, potentially offering greater stability and reducing expression-dependent variability. Furthermore, their core genomic localization minimizes the risk of horizontal transfer compared to mobile genetic elements like *bla_OXA-51-like_*. This combination of high specificity, ultra-sensitivity, and robust performance in clinical samples positions these novel targets as ideal candidates for developing next-generation molecular diagnostics, including multiplex assays or point-of-care platforms, to combat the significant threat posed by *A. baumannii* infections in healthcare environments.

Despite the promising results, this study has several limitations. The pan-genome analysis is based on 642 *A. baumannii* genomes, which is a considerable number, but with the continuous evolution of strains, there may be detection limitations in the future. Although the qPCR based on *ureE* shows excellent sensitivity, its performance in complex multi-species background samples still requires further research. Finally, the potential changes in the function of these target genes in *A. baumannii* in different environments need to be clarified, as this could affect the long-term diagnostic stability.

## 5 Conclusion

In conclusion, the nucleic acid detection method established based on new targets is an effective tool for the rapid and specific detection of *A. baumannii*, demonstrating its suitability for exploring pathogen differences. Therefore, this method can serve as an effective alternative for the routine diagnosis of clinical *A. baumannii* infections. Future studies should focus on expanding clinical sample validation, development of multiplex PCR or qPCR assays/panels based on these targets, and combined with new technologies such as CRISPR-Cas to develop ultra-sensitive and rapid POCT methods. These novel targets will be crucial for developing improved high-throughput detection methods, enhancing our capacity for effective treatment and prevention of transmission in healthcare settings.

## Data Availability

The original contributions presented in the study are included in the article/Supplementary material, further inquiries can be directed to the corresponding author/s.

## Footnotes

^1^ https://www.ncbi.nlm.nih.gov/datasets/genome/

^2^ https://www.ebi.ac.uk/ebisearch
