# Supplementary material for: Rapid identification of Acinetobacter baumannii using novel specific molecular targets derived from pan-genome analysis and its clinical application
Source: Front Microbiol. 2025 Oct 29;16:1669811. doi: 10.3389/fmicb.2025.1669811 (PMC12605139; doi:10.3389/fmicb.2025.1669811)
Supplement: Supplementary file 1 [file Data_Sheet_1.ZIP › Supplementary Material2.docx]

**Supplementary Material**

**Rapid identification of *Acinetobacter baumannii* using novel specific molecular targets derived from pan-genome analysis and its clinical application**

Shuang Chen^a^, Huayang Wang^b^, Chen Ju^b^, Pengliang Zhang^a^, YongQiang Ren^a^, Yaxuan Chen^b^, Xiaoqi Yi^b^, JianYing Zhang^a,*^, Shenghang Zhang^c,*^, Xinran Xiang^b,c*^

*^a^ The First Affiliated Hospital of Henan University of Science and Technology, Luoyang 471023, China*

*^b^ School of Life Science, Huaiyin Normal University, Huai’an 223300, China*

*^c^ Fujian Key Laboratory of Aptamers Technology, Fuzhou General Actual Medical School (the 900th Hospital), Fujian Medical University, Fuzhou 350001, China*

*Corresponding author:

JianYing Zhang

The First Affiliated Hospital of Henan University of science and Technology

Email: [zhangyingjian@haust.e du.cn](mailto:zhangyingjian@haust.edu.cn)

Shenghang Zhang

Fujian Key Laboratory of Aptamers Technology, Fuzhou General Actual Medical School (the 900th Hospital), Fujian Medical University

Email: fzzyyzsh@126.com

Xinran Xiang

Huaiyin Normal University

Fujian Key Laboratory of Aptamers Technology, Fuzhou General Actual Medical School (the 900th Hospital), Fujian Medical University

E-mail: [xiangxr2022@163.com](mailto:xiangxr2022@163.com)

**Table S1** Bacterial strains use in this study.

| Target strain | | | | | |
| --- | --- | --- | --- | --- | --- |
|  | No. | Bacterial species | Strain ID | Source of isolation | Location |
|  | 1 | *Acinetobacter baumannii* | ATCC 19606 | Reference strain | / |
|  | 2 | *Acinetobacter baumannii* | AB001 | Sputum | Hospital |
|  | 3 | *Acinetobacter baumannii* | AB002 | Sputum | Hospital |
|  | 4 | *Acinetobacter baumannii* | AB003 | Sputum | Hospital |
|  | 5 | *Acinetobacter baumannii* | AB004 | Sputum | Hospital |
|  | 6 | *Acinetobacter baumannii* | AB005 | Sputum | Hospital |
|  | 7 | *Acinetobacter baumannii* | AB006 | Sputum | Hospital |
|  | 8 | *Acinetobacter baumannii* | AB007 | Sputum | Hospital |
|  | 9 | *Acinetobacter baumannii* | AB008 | Sputum | Hospital |
|  | 10 | *Acinetobacter baumannii* | AB009 | Sputum | Hospital |
|  | 11 | *Acinetobacter baumannii* | AB010 | Sputum | Hospital |
|  | 12 | *Acinetobacter baumannii* | AB011 | Sputum | Hospital |
|  | 13 | *Acinetobacter baumannii* | AB012 | Sputum | Hospital |
|  | 14 | *Acinetobacter baumannii* | AB013 | Sputum | Hospital |
|  | 15 | *Acinetobacter baumannii* | AB014 | Sputum | Hospital |
|  | 16 | *Acinetobacter baumannii* | AB015 | Sputum | Hospital |
|  | 17 | *Acinetobacter baumannii* | AB016 | Sputum | Hospital |
|  | 18 | *Acinetobacter baumannii* | AB017 | Sputum | Hospital |
|  | 19 | *Acinetobacter baumannii* | AB018 | Sputum | Hospital |
|  | 20 | *Acinetobacter baumannii* | AB019 | Alveolar lavage fluid | Hospital |
|  | 21 | *Acinetobacter baumannii* | AB020 | Sputum | Hospital |
|  | 22 | *Acinetobacter baumannii* | AB021 | Drainage fluid | Hospital |
|  | 23 | *Acinetobacter baumannii* | AB022 | Sputum | Hospital |
|  | 24 | *Acinetobacter baumannii* | AB023 | Sputum | Hospital |
|  | 25 | *Acinetobacter baumannii* | AB024 | Alveolar lavage fluid | Hospital |
|  | 26 | *Acinetobacter baumannii* | AB025 | Wound secretion | Hospital |
|  | 27 | *Acinetobacter baumannii* | AB026 | Sputum | Hospital |
|  | 28 | *Acinetobacter baumannii* | AB027 | Sputum | Hospital |
|  | 29 | *Acinetobacter baumannii* | AB028 | Sputum | Hospital |
|  | 30 | *Acinetobacter baumannii* | AB029 | Sputum | Hospital |
|  | 31 | *Acinetobacter baumannii* | AB030 | Alveolar lavage fluid | Hospital |
|  | 32 | *Acinetobacter baumannii* | AB031 | Alveolar lavage fluid | Hospital |
|  | 33 | *Acinetobacter baumannii* | AB032 | Sputum | Hospital |
|  | 34 | *Acinetobacter baumannii* | AB033 | Sputum | Hospital |
|  | 35 | *Acinetobacter baumannii* | AB034 | Alveolar lavage fluid | Hospital |
|  | 36 | *Acinetobacter baumannii* | AB035 | Sputum | Hospital |
|  | 37 | *Acinetobacter baumannii* | AB036 | Sputum | Hospital |
|  | 38 | *Acinetobacter baumannii* | AB037 | Drainage fluid | Hospital |
|  | 39 | *Acinetobacter baumannii* | AB038 | Sputum | Hospital |
|  | 40 | *Acinetobacter baumannii* | AB039 | Alveolar lavage fluid | Hospital |
|  | 41 | *Acinetobacter baumannii* | AB040 | Sputum | Hospital |
|  | 42 | *Acinetobacter baumannii* | AB041 | Sputum | Hospital |
|  | 43 | *Acinetobacter baumannii* | AB042 | Sputum | Hospital |
|  | 44 | *Acinetobacter baumannii* | AB043 | Sputum | Hospital |
|  | 45 | *Acinetobacter baumannii* | AB044 | Alveolar lavage fluid | Hospital |
|  | 46 | *Acinetobacter baumannii* | AB045 | Alveolar lavage fluid | Hospital |
|  | 47 | *Acinetobacter baumannii* | AB046 | Sputum | Hospital |
|  | 48 | *Acinetobacter baumannii* | AB047 | Sputum | Hospital |
|  | 49 | *Acinetobacter baumannii* | AB048 | Alveolar lavage fluid | Hospital |
|  | 50 | *Acinetobacter baumannii* | AB049 | Sputum | Hospital |
|  | 51 | *Acinetobacter baumannii* | AB050 | Sputum | Hospital |
|  | 52 | *Acinetobacter baumannii* | AB051 | Alveolar lavage fluid | Hospital |
|  | 53 | *Acinetobacter baumannii* | AB052 | Sputum | Hospital |
|  | 54 | *Acinetobacter baumannii* | AB053 | Sputum | Hospital |
|  | 55 | *Acinetobacter baumannii* | AB054 | Sputum | Hospital |
|  | 56 | *Acinetobacter baumannii* | AB055 | Sputum | Hospital |
|  | 57 | *Acinetobacter baumannii* | AB056 | Sputum | Hospital |
|  | 58 | *Acinetobacter baumannii* | AB057 | Drainage fluid | Hospital |
|  | 59 | *Acinetobacter baumannii* | AB058 | Sputum | Hospital |
|  | 60 | *Acinetobacter baumannii* | AB059 | Alveolar lavage fluid | Hospital |
|  | 61 | *Acinetobacter baumannii* | AB060 | Sputum | Hospital |
|  | 62 | *Acinetobacter baumannii* | AB061 | Sputum | Hospital |
|  | 63 | *Acinetobacter baumannii* | AB062 | Sputum | Hospital |
|  | 64 | *Acinetobacter baumannii* | AB063 | Sputum | Hospital |
|  | 65 | *Acinetobacter baumannii* | AB064 | Wound secretion | Hospital |
|  | 66 | *Acinetobacter baumannii* | AB065 | Alveolar lavage fluid | Hospital |
|  | 67 | *Acinetobacter baumannii* | AB066 | Sputum | Hospital |
|  | 68 | *Acinetobacter baumannii* | AB067 | Sputum | Hospital |
|  | 69 | *Acinetobacter baumannii* | AB068 | Alveolar lavage fluid | Hospital |
|  | 70 | *Acinetobacter baumannii* | AB069 | Sputum | Hospital |
|  | 71 | *Acinetobacter baumannii* | AB070 | Sputum | Hospital |
|  | 72 | *Acinetobacter baumannii* | AB071 | Sputum | Hospital |
|  | 73 | *Acinetobacter baumannii* | AB072 | Sputum | Hospital |
|  | 74 | *Acinetobacter baumannii* | AB073 | Sputum | Hospital |
|  | 75 | *Acinetobacter baumannii* | AB074 | Alveolar lavage fluid | Hospital |
|  | 76 | *Acinetobacter baumannii* | AB075 | Sputum | Hospital |
|  | 77 | *Acinetobacter baumannii* | AB076 | Puncture fluid | Hospital |
|  | 78 | *Acinetobacter baumannii* | AB077 | Sputum | Hospital |
|  | 79 | *Acinetobacter baumannii* | AB078 | Alveolar lavage fluid | Hospital |
|  | 80 | *Acinetobacter baumannii* | AB079 | Alveolar lavage fluid | Hospital |
|  | 81 | *Acinetobacter baumannii* | AB080 | Sputum | Hospital |
|  | 82 | *Acinetobacter baumannii* | AB081 | Sputum | Hospital |
|  | 83 | *Acinetobacter baumannii* | AB082 | Bile | Hospital |
|  | 84 | *Acinetobacter baumannii* | AB083 | Catheter | Hospital |
|  | 85 | *Acinetobacter baumannii* | AB084 | Sputum | Hospital |
|  | 86 | *Acinetobacter baumannii* | AB085 | Sputum | Hospital |
|  | 87 | *Acinetobacter baumannii* | AB086 | Sputum | Hospital |
|  | 88 | *Acinetobacter baumannii* | AB087 | Wound secretion | Hospital |
|  | 89 | *Acinetobacter baumannii* | AB088 | Blood | Hospital |
|  | 90 | *Acinetobacter baumannii* | AB089 | Sputum | Hospital |
|  | 91 | *Acinetobacter baumannii* | AB090 | Alveolar lavage fluid | Hospital |
|  | 92 | *Acinetobacter baumannii* | AB091 | Sputum | Hospital |
|  | 93 | *Acinetobacter baumannii* | AB092 | Sputum | Hospital |
|  | 94 | *Acinetobacter baumannii* | AB093 | Alveolar lavage fluid | Hospital |
|  | 95 | *Acinetobacter baumannii* | AB094 | Sputum | Hospital |
|  | 96 | *Acinetobacter baumannii* | AB095 | Sputum | Hospital |
|  | 97 | *Acinetobacter baumannii* | AB096 | Sputum | Hospital |
|  | 98 | *Acinetobacter baumannii* | AB097 | Sputum | Hospital |
|  | 99 | *Acinetobacter baumannii* | AB098 | Sputum | Hospital |
|  | 100 | *Acinetobacter baumannii* | AB099 | Urine | Hospital |
|  | 101 | *Acinetobacter baumannii* | AB100 | Sputum | Hospital |
|  | 102 | *Acinetobacter baumannii* | AB101 | Sputum | Hospital |
|  | 103 | *Acinetobacter baumannii* | AB102 | Alveolar lavage fluid | Hospital |
|  | 104 | *Acinetobacter baumannii* | AB103 | Sputum | Hospital |
|  | 105 | *Acinetobacter baumannii* | AB104 | Alveolar lavage fluid | Hospital |
|  | 106 | *Acinetobacter baumannii* | AB105 | Sputum | Hospital |
|  | 107 | *Acinetobacter baumannii* | AB106 | Sputum | Hospital |
|  | 108 | *Acinetobacter baumannii* | AB107 | Alveolar lavage fluid | Hospital |
|  | 109 | *Acinetobacter baumannii* | AB108 | Alveolar lavage fluid | Hospital |
|  | 110 | *Acinetobacter baumannii* | AB109 | Sputum | Hospital |
|  | 111 | *Acinetobacter baumannii* | AB110 | Sputum | Hospital |
|  | 112 | *Acinetobacter baumannii* | AB111 | Sputum | Hospital |
|  | 113 | *Acinetobacter baumannii* | AB12 | Sputum | Hospital |
|  | 114 | *Acinetobacter baumannii* | AB13 | Sputum | Hospital |
|  | 115 | *Acinetobacter baumannii* | AB114 | Alveolar lavage fluid | Hospital |
|  | 116 | *Acinetobacter baumannii* | AB115 | Throat swab | Hospital |
|  | 117 | *Acinetobacter baumannii* | AB116 | Sputum | Hospital |
|  | 118 | *Acinetobacter baumannii* | AB117 | Alveolar lavage fluid | Hospital |
|  | 119 | *Acinetobacter baumannii* | AB118 | Sputum | Hospital |
|  | 120 | *Acinetobacter baumannii* | AB119 | Alveolar lavage fluid | Hospital |
|  | 121 | *Acinetobacter baumannii* | AB120 | Sputum | Hospital |
|  | 122 | *Acinetobacter baumannii* | AB121 | Sputum | Hospital |
|  | 123 | *Acinetobacter baumannii* | AB122 | Sputum | Hospital |
|  | 124 | *Acinetobacter baumannii* | AB123 | Sputum | Hospital |
|  | 125 | *Acinetobacter baumannii* | AB124 | Alveolar lavage fluid | Hospital |
|  | 126 | *Acinetobacter baumannii* | AB125 | Sputum | Hospital |
|  | 127 | *Acinetobacter baumannii* | AB126 | Sputum | Hospital |
|  | 128 | *Acinetobacter baumannii* | AB127 | Sputum | Hospital |
|  | 129 | *Acinetobacter baumannii* | AB128 | Sputum | Hospital |
|  | 130 | *Acinetobacter baumannii* | AB129 | Sputum | Hospital |
|  | 131 | *Acinetobacter baumannii* | AB130 | Alveolar lavage fluid | Hospital |
|  | 132 | *Acinetobacter baumannii* | AB131 | Puncture fluid | Hospital |
|  | 133 | *Acinetobacter baumannii* | AB132 | Alveolar lavage fluid | Hospital |
|  | 134 | *Acinetobacter baumannii* | AB133 | Alveolar lavage fluid | Hospital |
|  | 135 | *Acinetobacter baumannii* | AB134 | Urine | Hospital |
|  | 136 | *Acinetobacter baumannii* | AB135 | Sputum | Hospital |
|  | 137 | *Acinetobacter baumannii* | AB136 | Sputum | Hospital |
|  | 138 | *Acinetobacter baumannii* | AB137 | Alveolar lavage fluid | Hospital |
|  | 139 | *Acinetobacter baumannii* | AB138 | Sputum | Hospital |
|  | 140 | *Acinetobacter baumannii* | AB139 | Throat swab | Hospital |
|  | 141 | *Acinetobacter baumannii* | AB140 | Sputum | Hospital |
|  | 142 | *Acinetobacter baumannii* | AB141 | Blood | Hospital |
|  | 143 | *Acinetobacter baumannii* | AB142 | Urine | Hospital |
|  | 144 | *Acinetobacter baumannii* | AB143 | Sputum | Hospital |
|  | 145 | *Acinetobacter baumannii* | AB144 | Sputum | Hospital |
|  | 146 | *Acinetobacter baumannii* | AB145 | Sputum | Hospital |
|  | 147 | *Acinetobacter baumannii* | AB146 | Sputum | Hospital |
|  | 148 | *Acinetobacter baumannii* | AB147 | Sputum | Hospital |
|  | 149 | *Acinetobacter baumannii* | AB148 | Alveolar lavage fluid | Hospital |
|  | 150 | *Acinetobacter baumannii* | AB149 | Sputum | Hospital |
|  | 151 | *Acinetobacter baumannii* | AB150 | Sputum | Hospital |
|  | 152 | *Acinetobacter baumannii* | AB151 | Drainage fluid | Hospital |
|  | 153 | *Acinetobacter baumannii* | AB152 | Sputum | Hospital |
|  | Non-target strains (including standard strains) | | | | |
|  | No. | Bacterial species | Strain ID | Source of isolation | Location |
|  |  | *Acinetobacter calcoaceticus* | CI001 | Blood | Hospital |
|  | 1 | *Acinetobacter lwoffi* | CI002 | Bile | Hospital |
|  | 2 | *Acinetobacter haemolytius* | CI003 | Cerebrospinal fluid | Hospital |
|  | 3 | *Acinetobacter junii* | CI004 | Urine | Hospital |
|  | 5 | *Acinetobacter johnsonii* | CI005 | Urine | Hospital |
|  | 6 | *Acinetobacter radioresistens* | CI006 | Seroperitoneum | Hospital |
|  | 7 | *Staphylococcus warneri* | CI007 | Blood | Hospital |
|  | 8 | *Streptococcus agalactiae* | CI008 | Blood | Hospital |
|  | 9 | *Staphylococcus capitis* | CI009 | Cervical secretion | Hospital |
|  | 10 | *Staphylococcus saprophyticus* | CI010 | Blood | Hospital |
|  | 11 | *Enterococcus gallinarum* | CI011 | Bile | Hospital |
|  | 12 | *Streptococcus constellatus subsp.constellatus* | CI012 | Bile | Hospital |
|  | 13 | *Enterococcus faecium* | CI013 | Puncture fluid | Hospital |
|  | 14 | *Streptococcus gallolyticus* | CI014 | Seroperitoneum | Hospital |
|  | 15 | *Staphylococcus saprophyticus* | CI015 | Blood | Hospital |
|  | 16 | *Cupriavidus* | CI016 | Drainage fluid | Hospital |
|  | 17 | *Listeria monocytogenes* | ATCC 19115 | Reference strain | / |
|  | 18 | *Bacillus cereus* | ATCC 11778 | Reference strain | / |
|  | 19 | *Salmonella Enteritidis* | ATCC 13076 | Reference strain | / |
|  | 20 | *Cronobacter sakazakii* | ATCC 29544 | Reference strain | / |
|  | 21 | *Staphylococcus aureus* | ATCC 29213 | Reference strain | / |
|  | 22 | *Escherichia coli* O157:H7 | ATCC 700728 | Reference strain | / |

**Table S2** PCR and qPCR primer sets used for *A. baumannii* molecular targets.

| Detection method | Target | AB ID NO. | Primer | Sequence (5’ to 3’) | Product size (bp) | Primer position (bp) | Primer size (bp) |
| --- | --- | --- | --- | --- | --- | --- | --- |
| PCR | *outO* | 1 | NO.1F | AATTATTGCCTGATCGCTTT | 192 | 464 | 20 |
|  |  |  | NO.1R | GCAATTTAAAGTCGCCGTA |  | 637 | 19 |
|  |  | 2 | NO.2F | ATCAGTATTCGCTATCCAG | 120 | 316 | 19 |
|  |  |  | NO.2R | TATCAGTACCCATGTGAGG |  | 417 | 19 |
|  |  | 3 | NO.3F | AAGAATGCCAAATGTTACTCA | 161 | 140 | 21 |
|  |  |  | NO.3R | GCCACATTTTCCTCTTAACAC |  | 280 | 21 |
|  | *ureE* | 4 | NO.4F | TGTATTACGTGTCGATGCCAA | 111 | 192 | 21 |
|  |  |  | NO.4R | ATTAATGGCACATGTCGGTT |  | 283 | 20 |
|  |  | 5 | NO.5F | CTAACGTTTGATACCCGCCAA | 135 | 64 | 21 |
|  |  |  | NO.5R | TAATACATCCCCTTCTTGCGTTG |  | 176 | 23 |
|  |  | 6 | NO.6F | TAACGTTTGATACCCGCCAA | 299 | 65 | 20 |
|  |  |  | NO.6R | ACCTTCTACCATTTCTGCAAG |  | 343 | 21 |
|  | *rplY* | 7 | NO.7F | TTCCAGCTATCATCTACGG | 201 | 89 | 19 |
|  |  |  | NO.7R | GTTTGAAGTCAGCGTGCAT |  | 271 | 19 |
|  |  | 8 | NO.8F | TTCCAGCTATCATCTACGG | 173 | 89 | 19 |
|  |  |  | NO.8R | TTTAGCTGGGTGACGTTG |  | 244 | 18 |
|  |  | 9 | NO.9F | ACTTTAGAACTTCGTGAGCTTG | 158 | 133 | 22 |
|  |  |  | NO.9R | CGTTTGAAGTCAGCGTGCAT |  | 271 | 20 |
|  | *bioF* | 10 | NO.10F | GCTCGATCATTTTGCTACAGA | 1147 | 9 | 21 |
|  |  |  | NO.10R | CAGGAAACTCACCAATGCTT |  | 1136 | 20 |
|  |  | 11 | NO.11F | TCAATTGGCGTACGTGGA | 209 | 640 | 18 |
|  |  |  | NO.11R | CATGCCATAACAATCGGAGG |  | 829 | 20 |
|  |  | 12 | NO.12F | CTCGATCATTTTGCTACAGA | 211 | 10 | 20 |
|  |  |  | NO.12R | AACTAAAATAGCTGCGTTC |  | 202 | 19 |
|  | *menH_3* | 13 | NO.13F | AACGATTCACAAAACACCGAT | 174 | 156 | 21 |
|  |  |  | NO.13R | GTTCCAGCAGAAATTCCGGTA |  | 309 | 21 |
|  |  | 14 | NO.14F | GAACATTAGAGCTTCCCGAT | 160 | 326 | 20 |
|  |  |  | NO.14R | CCCATTAAAATCATACGGTCCA |  | 464 | 22 |
|  |  | 15 | NO.15F | AACGATTCACAAAACACCGAT | 331 | 156 | 21 |
|  |  |  | NO.15R | ACCCATTAAAATCATACGGTCCA |  | 464 | 23 |
|  | *hemW* | 16 | NO.16F | TGGCAAAGTAACCCGACCA | 242 | 825 | 19 |
|  |  |  | NO.16R | TCGAACGCAATGAAGTAAGCA |  | 1046 | 21 |
|  |  | 17 | NO.17F | CACTAGAACATGACCCGTTT | 732 | 335 | 20 |
|  |  |  | NO.17R | TCGAACGCAATGAAGTAAGCA |  | 1046 | 21 |
|  |  | 18 | NO.18F | CACTAGAACATGACCCGTTT | 479 | 335 | 20 |
|  |  |  | NO.18R | ACCAATCGCAAGATAATCACC |  | 793 | 21 |
|  | *paaF_1* | 19 | NO.19F | CTTAGCCATAAATCGCCCAG | 138 | 69 | 20 |
|  |  |  | NO.19R | GTAAAGTCATGTTCGGCACCA |  | 186 | 21 |
|  |  | 20 | NO.20F | CTCACCTTAGCCATAAATCGC | 143 | 64 | 21 |
|  |  |  | NO.20R | GTAAAGTCATGTTCGGCACCA |  | 186 | 21 |
|  |  | 21 | NO.21F | CTGCCAGATTATCGAAGCC | 495 | 302 | 19 |
|  |  |  | NO.21R | TAAACTGTGAAAAGTCCGGTT |  | 776 | 21 |
|  | *smpB* | 22 | NO.22F | GATTCAACCACTCCTTTCTGC | 157 | 210 | 21 |
|  |  |  | NO.22R | ATGACCTTTCCAGTAACATGC |  | 346 | 21 |
|  |  | 23 | NO.23F | TGCTCAGATTCAACCACTCC | 163 | 204 | 20 |
|  |  |  | NO.23R | ATGACCTTTCCAGTAACATGC |  | 346 | 21 |
|  |  | 24 | NO.24F | GATTCAACCACTCCTTTCTGC | 250 | 210 | 21 |
|  |  |  | NO.24R | TGCTTTATCACGTTGCCAGT |  | 440 | 20 |
|  | *ppaX* | 25 | NO.25F | CACTATATTGCCAACTCGAC | 133 | 226 | 20 |
|  |  |  | NO.25R | CAATCACACGGTCTAATCCAC |  | 338 | 21 |
|  |  | 26 | NO.26F | CGCAAAGAGTATTATCGGTT | 232 | 126 | 20 |
|  |  |  | NO.26R | AATCACACGGTCTAATCCAC |  | 338 | 20 |
|  |  | 27 | NO.27F | TCACTATATTGCCAACTCGAC | 309 | 225 | 21 |
|  |  |  | NO.27R | ATACCTAAACGCTGAGCCAT |  | 514 | 20 |
| qPCR | *outO* | 28 | NO.28F | ACTGCACTTTATATTGCTG | 126 | 37 | 19 |
|  |  |  | NO.28R | ATTGAGTAACATTTGGCAT |  | 144 | 19 |
|  | *ureE* | 29 | NO.29F | ACGGTTGAGCTAACGTTT | 141 | 55 | 18 |
|  |  |  | NO.29R | TACATCCCCTTCTTGCGTTG |  | 176 | 20 |
|  | *rplY* | 30 | NO.30F | GGCAAACTTCGTATTAAACGCTCA | 288 | 3 | 24 |
|  |  |  | NO.30R | CGTTTGAAGTCAGCGTGCAT |  | 271 | 20 |
|  | *bioF* | 31 | NO.31F | ATTTAACCGCTTTAGTCCAG | 121 | 572 | 20 |
|  |  |  | NO.31R | ACATTATATTGTTCGGCACAC |  | 672 | 21 |
|  | *menH_3* | 32 | NO.32F | CCCGGTGATTTTAATCGACT | 144 | 249 | 20 |
|  |  |  | NO.32R | TTTTGAATTCCGACGATATCTAGC |  | 369 | 24 |
|  | *hemW* | 33 | NO.33F | GCTGCTTAACTTTGAAGATGACT | 97 | 282 | 23 |
|  |  |  | NO.33R | AATTCCTGCTTCTAAATAGCCT |  | 357 | 22 |
|  | *paaF_1* | 34 | NO.34F | GAAGCCGCTTATTATTGCAGT | 66 | 315 | 21 |
|  |  |  | NO.34R | GCCTGTAATAAAATCGTAACACC |  | 358 | 23 |
|  | *smpB* | 35 | NO.35F | TAGTAAAGAAACATAATGGCGGAA | 144 | 17 | 24 |
|  |  |  | NO.35R | CTGTCAAACTCATACGACC |  | 142 | 19 |
|  | *ppaX* | 36 | NO.36F | CCGTTCAAAACCAGACCC | 129 | 411 | 18 |
|  |  |  | NO.36R | CGAGGCATACCTAAACGC |  | 522 | 18 |

**Table S3** Sequences of *A. baumannii* molecular targets.

| SEQ ID NO. | Target | Nucleic acid sequence |
| --- | --- | --- |
| 1 | *outO* | ATGCAAGACATCATTGCGTATTTTATTCAAAACTTAACTGCACTTTATATTGCTGTTGCACTCGTGAGCCTATGTATCGGTAGCTTTCTTAATGTAGTAATTTACCGCACGCCAAGAATGATGGAGCAAGATTGGCAGCAAGAATGCCAAATGTTACTCAATCCTGAGCAACCAATTATTGATCATGAGAGGTTAACTTTAAATAAGCCTGCTTCATCGTGCCCTGCATGTCAGCAACCGATCCGTTGGTATCAAAATATTCCTGTTATAAGCTGGCTTGTGTTAAGAGGAAAATGTGGCCATTGCCAACACCCGATCAGTATTCGCTATCCAGCCATCGAACTACTCACCATGCTATGTTCATTAGTAGTAGTCATGGTATTTGGCCCAACCATACAAATGCTTTTTGGACTCATCCTCACATGGGTACTGATAGCCCTTACCTTTATTGATTTCGATACACAATTATTGCCTGATCGCTTTACCCTACCTTTAGCTGCGCTCGGCTTAGGTATTAATACCTTTAATATTTATACCTCACCCAACTCAGCCATTTGGGGTTATCTCATTGGTTTCCTATGTCTTTGGATTGTCTATTACTTATTTAAAGTGATCACTGGCAAAGAAGGTATGGGCTACGGCGACTTTAAATTGCTTGCAGCATTAGGAGCATGGATGGGGCCATTGATGCTGCCGTTAATTGTGTTATTGTCATCGTTAATTGGCGCAATCATTGGCATCATTTTATTAAAATTAAGAAATGACAATCAGCCTTTTGCTTTTGGGCCATATATTGCCATTGCTGGTTGGGTTGCCTTTTTATGGGGTGATCAGATTATGAAAATTTATTTGGGAGGTTAA |
| 2 | *ureE* | ATGAAAATTTACACCCAACGTCTTGAAGATATTTCTCCTGATCAGGCATTTGAAACGGTTGAGCTAACGTTTGATACCCGCCAAAAATCACGTTTTCGAGCGGCTTTAGCGAGTGGCGTAGATATTGGTGCGGATTTGCCACGTACCGGCATTTTGCGTAGTGGTTCATATATTGCAACGCAAGAAGGGGATGTATTACGTGTCGATGCCAAACCTGAACGGCTAATGAAAGTCACTGCTCAGACTGAATTTGATTTGCTTAAAGCGGCTTATCATTTAGGTAACCGACATGTGCCATTAATGCTGACACCAACAGCTTTATATTTTGAGCCTGATCATGTGCTTGCAGAAATGGTAGAAGGTTTGGGACTTACTGTGACAGAAACCGATCATCCATTTGAACCTGAAAGCGGTGCCTATGCACAGCATAGCCATGACCATCGTTTAAGTCCAATCAAAGTTTTACACCATGTCCACTCATAA |
| 3 | *rplY* | ATGGCAAACTTCGTATTAAACGCTCAAGCGCGTGCTGAAGACAAACAAGGGAAAGGTGCGAGCCGCCGCCTTCGTCGCGAATCTTTAGTTCCAGCTATCATCTACGGTGGTAACGCTGAGCCTGTAGCAGTTACTTTAGAACTTCGTGAGCTTGTAAAAGCTTTAGAAAGCAACGCTTTCTTTGAAGAAGTTGTTGAAATCAAAGTAGGTGACAAAGTTGAAAACGTTAAAATCCAAGCGTTACAACGTCACCCAGCTAAAAACACTCCTATGCACGCTGACTTCAAACGCGCATAA |
| 4 | *bioF* | ATGTCATTGCTCGATCATTTTGCTACAGAGTTAGATGAGTTAAAACGACAAGGTAATTTCAGACAATTTACCCAAAATGTGCAGCATGGCCGTTTTATTACGATTCAAAATAAAACGATGCTCAACTTAGCTTCAAATGACTATCTTGGGTTGGCGGCTGATATTGATCTTCGCCAAGAATTTTTAGATTACTATCCATTAGAACGCAGCTATTTTAGTTCTTCATCTTCACGTTTATTAACGGGCAATTTTGATGAATATGAACAGTTGGAAAATAGCCTAAGCCAAGCATTTGGTGGTCGGGCGGCTCTATTATTCAATAGTGGCTACCATATGAATATTGGTATTTTGCCCGCTGTTGCTGATAGTAAAACTTTAATTCTGGCAGATAAGTTAGTTCATGCCAGTATGATTGATGGGATTCGTTTGTCTGGTGCCCAGTATGTGCGCTATCGACATAATGATTTTCAGCATTTAGAGCAACTTCTACAAAAGTATCATGATGATGACCAGATTGAACGAATTATTGTGGTCACGGAAAGTATTTTTAGTATGGATGGCGATGAAACAGATTTAACCGCTTTAGTCCAGCTTAAAAAACGCTTTGCTAAAACCATGTTATATGTCGATGAAGCGCATTCAATTGGCGTACGTGGACAGCAAGGTTTAGGGTGTGCCGAACAATATAATGTAATTCAGGAAATTGATTTTCTAGTTGGTACCTTGGGTAAAGCGCTAGCCGCAGTCGGTGGTTATATTATTTGCCATCCAATTATTAAAGATTATCTCATTAATAAAATGCGTCCGCTTATTTTTAGTACCGCACAACCTCCGATTGTTATGGCATGGGCAAATTTTATTTTTAAAAAAGTATTAATGGCTCAATCTCAACGTGAACATTTAAAAAATATTAGCCAATATCTTCAACAAGCAGTGGTGCAAAAGGGGTATGTGAGTCCATCGACCAGCCATATTATTCCAGTGATTGTGGGAGAGAGTCAGGCTACTATCGATAAGGCGAAACAGGTTCAGGAAAGCGGTTTTTATGCCATGCCTGTACGTCCACCAACAGTACCTAAAAATAGTTCGCGTTTGCGCATTTCCTTAACATCAATGGTTGAGCAACATGAACTTGAAGCATTGGTGAGTTTCCTGTGA |
| 5 | *menH_3* | ATGAATTCCATTATTCAAATGGATTCGGAGATAAACCAAGCTTATGCCGGTTTTGGGTTTTTCGATATCTACCATAGAGATAGTTTTAAACAGCCAGCTCGTACCACATGGATTGACGGTTGGAAAATTGAATATATGGCGATTGCAGACCCACAAACGATTCACAAAACACCGATTGTGATTGTTGGCGGTGCTTTTCAAAATTTTAACTCGTATAAATATTGTGTTGAACAACTATTTGAAAGTGGCCCGGTGATTTTAATCGACTTGCCATCTATGGGCGCAAATCAACAAATTACCAATCGAGATACCGGAATTTCTGCTGGAACATTAGAGCTTCCCGATTTGTCGGAAATGTTAGGACGTTGGCTAGATATCGTCGGAATTCAAAAAGTTTCTGTAATGGGAATGTCGTTGGGTTCAGTTGTTGCTTCATGTTTTGCTTATCATCGTCCAGATTTGATGGACCGTATGATTTTAATGGGTGTTATGCAAAAGACCCGTAAAAGCTGGCGGATGATTCTGGAAGAGTCGCTCAAACTCATGCAAGAAAATCGTATGGAAGAGTTTGGGCAAGCGGTAATTTTATATTTGGTGAATCATGCCAAATTAGATAAAACACGTATGTCGCCTACAGCTAAAAAATTATTTTTTAGGCAAATGGCAGAATTTACGGGTACTGAAAGAGAACGTTATGAAATTAACTGTAATCGCCTTTTGCGTTTAACAGATGTACCTATTCCAGAATGTAAGACACTGGTCGCAGCAGGGCAGTACGATAGTTTTACTTTGCCTCATGAAAATGCCAATTTTGCTTTGCAATGTCCAGATATGGAATTTGCTCTGATTGCTAACGCCGACCATGTGCCGCAGCTCCAGCGCCGTAAAGAAACTATGAGTTTGTTCACTTCATTTTTGAAAGGTGAACCTATCCAAAATCTGGATGGCATTATTCCAATGACGCGTGAGCAAATGCAAAACATGGAACGCCGAGGTGAAGAGCGTATACCAGTTCTCCAGCCTAAAACGAAACTGAGCCACCGTGAATGTGAAACAGAGGTGCCAGTGATGATTATTGATGTGACTTTTTTTGGAATGTATTTGAAATTAGATGATGTAGCACAGCTTGAGTTTGTAAATGAACATCCACGTGATTTGGCTTTACATTTAGAAGATGAAGAGGGCGCTTTTTCGATTGAGTGTTTAATCTTTGAAGCTACTGAACAAGGAGTCCGTGCCTTATTTAAACATGGTAGTTTTGAACTTGCTGACCGTTTAAGCCGCTTCATTACTCGCCAGAAACAAGCAGCTTAA |
| 6 | *hemW* | TTGGCCGTATTAAATCCTGCTTCTGTTCCTTTATCTTTATATATCCACATGCCTTGGTGCGTGCGTAAATGTCCCTATTGCGACTTTAACTCACATGCGGTGCCTGATGGTGCGCTTTCGGCAGAACTCGAGCAAACTTATTTAAAAGCATTGGTAGCCGACTTTGAAACTCAAGTCGAGATGGCACAAGGACGTTCAATTCACAGTGTATTTATTGGTGGTGGAACACCTTCACTTATTTCTGCTAAAGGTTATGCATGGCTATTTGAACAGCTTAAATCGCTGCTTAACTTTGAAGATGACTGTGAAATTACTTTAGAAGCTAATCCGGGAACACTAGAACATGACCCGTTTGCAGGCTATTTAGAAGCAGGAATTAATCGTTTATCGATTGGTGTACAGACTTTTAATACCGATCACCTGCAAAAGCTTGGGCGTATTCATTCAGCCAACAATGCTATGGATGCAATTGAGCAGGCACGACAAGCAGGTTTTAAGCGAGTTAATGTCGATTTGATGCATGGCTTGCCAGAACAAACACTAGAGCAAGCACTTTATGATTTAAAGACGGCTGTAGAGCAGGGTGCAACACATATCAGCTGGTATCAACTGACCATTGAACCAAATACTGTATTTTTCCGAACACAACCTGTTTTACCTCAAGATGAGGTGTTAGAAGATATTCAAGAGCAAGGTGAAGCCTATCTAAAAGCAAATGGCTTTATCAATTATGAAGTGTCAGCTTGGCGTAAAGAGCAGCCTTCAGCTCATAATTTAAATTATTGGCAATTTGGTGATTATCTTGCGATTGGTGCAGGAGCACATGGCAAAGTAACCCGACCAGATGGTGTATATCGTTTTCAAAAGACTCGTTTACCGAAAGACTATTTGGCTAAGGTTCCAGCCGAACATTTGCAAATGAAAAAGATTGAAGCAGATGAACTACCTTTTGAGTTTATGATGAACGCTTTACGTTTAAATGATGGGGTAAAAGCTGAGTTATATGAACAGCGTACAGGCTTAAGTTTAGATGATTTAAATGATCTGCTTACTTCATTGCGTTCGAGAGAGTTATTGGTAGAGGATAGCGATCGTTTGGCTTGTACAGAGCAGGGGCATATTTTCTTAAACTCGGTACTCGAAGAGTTCTTATAA |
| 7 | *paaF_1* | ATGACACTTAGCTGTATTCAACAACCTCATCAACATTTACAAGCAAATTTAGAAGGTGGCGTACTCACCTTAGCCATAAATCGCCCAGAAGCAAAAAATGCATTATATGGCGAACTTTACTTATGGATTGCCAAAGCACTTGATGAAGCCGACCAAAATAAAGATGTACGAGTAATCGTTCTACGTGGTGCCGAACATGACTTTACCGCTGGTAATGACATGAAAGATTTTATGGGTTTTGTACAAAATCCGAATGCTGGTCCAGCAGGTCAAGTTCCTCCATTTGTACTCCTTAAATCTGCTGCCAGATTATCGAAGCCGCTTATTATTGCAGTAAAAGGCGTGGCAATTGGTATTGGTGTTACGATTTTATTACAGGCAGACCTCGTTTTTGCCGACAATACGGCCCTCTTTCAAATTCCTTTTGTTAGCCTTGGGCTTTCTCCTGAAGGTGGTGCAAGTCAATTATTGGTTAAACAAGCTGGTTATCACAAAGCAGCTGAACTACTGTTTACTGCCAAAAAGTTCAATGCAGAAACTGCTGTACAAGCTGGTTTAGTTAATGAAATCGTTGAAGATGCTTATGCCACTGCTCAGGCAACTGCCCAACACTTAACAGCTTTACCGCTTGCTTCATTAAAACAAACCAAAGCATTAATGAAACATGATTTAGATCAGATTATTGAATGTATTGACCATGAAGCAGAAATCTTTATGCAACGCGTTCAATCACCAGAAATGCTAGAAGCTGTGCAGGCATTTATGCAAAAACGTAAACCGGACTTTTCACAGTTTAATTAA |
| 8 | *smpB* | ATGGCGAAAGCAACAGTAGTAAAGAAACATAATGGCGGAACCATTGCACAAAACAAACGTGCCCGTCATGATTATTTTATCGAAGAAAAATTTGAAGCTGGCATGTCTTTACTAGGCTGGGAAGTAAAATCTTTACGTGCCGGTCGTATGAGTTTGACAGAAAGTTATGTCATTTTTAAAAATGGTGAAGCATTCTTATTTGGTGCTCAGATTCAACCACTCCTTTCTGCATCTACACATATTGTGCCGGAAGCTACACGTACACGAAAATTATTATTATCTCGTCGTGAACTTGAAAAGCTTATGGGTGCAGTGAACCAAAAAGGTTATTCGTGCGTTCCATTAGCATGTTACTGGAAAGGTCATCTGGTTAAGCTTGAAATTGCACTCGTGAAAGGTAAACAACTTCACGATAAACGAGCAACTGAAAAAGAACGTGACTGGCAACGTGATAAAGCACGTATATTTCATAAGTAA |
| 9 | *ppaX* | ATGAGTCTGAAAACCGAATTAGTGATTTTTGACTGGGATGGTACTTTATATAATTCTGTTGGTCAGATTGTTGCAAGTTTACAGCATGCGGCAGAAGAGCATAAGTTAACTCTGACTGATGAAGCCGCAAAGAGTATTATCGGTTTAGGTTTGCCGGAAGTGATGCAGACCTTATTTCCAGAAGTACCTGATTTGCATGATTCGATTTTAAAAGCATATGGCGATCACTATATTGCCAACTCGACAAATGATGCATGGTTTGAAGGTATTTCTGAACTACTTCATGACTTAAAAGCTCAAGGTTTAAAACTTGCAGTTGCGACGGGTAAAAACCGTCGTGGATTAGACCGTGTGATTGCAAAAACACAGAGTACTCATCTGTTTGATGTTACTCGAGCAGCCAATGAAACCCGTTCAAAACCAGACCCGCTCATGTTACAAGAAATCTTGACTGTAACTGGAGTAAGTGTCGAACAGGCAGTTATGATTGGTGATAGTAGTTATGACCTTGAAATGGCTCAGCGTTTAGGTATGCCTCGTATTGGGGTGGGCTATGGAGTGCACTCGGTCGAAGTTCTACAACAGTTCCAACCTTTAACCATTGCTAAAGATGTACCTGAGCTGCACAACTTTTTAAGAGAGTATGCAAAGTTATCTACTGTAGACGTAGCGTAA |

| Bacterial species | Source | PCR | | | | | | | | | qPCR | | | | | | | | |  |
| --- | --- | --- | --- | --- | --- | --- | --- | --- | --- | --- | --- | --- | --- | --- | --- | --- | --- | --- | --- | --- |
|  |  | *outO* | *ureE* | *rplY* | *bioF* | *menH_3* | *hemW* | *paaF_1* | *smpB* | *ppaX* | *outO* | *ureE* | *rplY* | *bioF* | *menH_3* | *hemW* | *paaF_1* | *smpB* | *ppaX* | |
| *Acinetobacter baumannii* | ATCC 19606 | + | + | + | + | + | + | + | + | + | + | + | + | + | + | + | + | + | + | |
| *Acinetobacter calcoaceticus* | Clinical isolate | - | - | - | - | - | - | - | - | - | - | - | - | - | - | - | - | - | - | |
| *Acinetobacter lwoffi* | Clinical isolate | - | - | - | - | - | - | - | - | - | - | - | - | - | - | - | - | - | - | |
| *Acinetobacter haemolytius* | Clinical isolate | - | - | - | - | - | - | - | - | - | - | - | - | - | - | - | - | - | - | |
| *Acinetobacter junii* | Clinical isolate | - | - | - | - | - | - | - | - | - | - | - | - | - | - | - | - | - | - | |
| *Acinetobacter johnsonii* | Clinical isolate | - | - | - | - | - | - | - | - | - | - | - | - | - | - | - | - | - | - | |
| *Acinetobacter radioresistens* | Clinical isolate | - | - | - | - | - | - | - | - | - | - | - | - | - | - | - | - | - | - | |
| *Staphylococcus warneri* | Clinical isolate | - | - | - | - | - | - | - | - | - | - | - | - | - | - | - | - | - | - | |
| *Streptococcus agalactiae* | Clinical isolate | - | - | - | - | - | - | - | - | - | - | - | - | - | - | - | - | - | - | |
| *Staphylococcus capitis* | Clinical isolate | - | - | - | - | - | - | - | - | - | - | - | - | - | - | - | - | - | - | |
| *Staphylococcus saprophyticus* | Clinical isolate | - | - | - | - | - | - | - | - | - | - | - | - | - | - | - | - | - | - | |
| *Enterococcus gallinarum* | Clinical isolate | - | - | - | - | - | - | - | - | - | - | - | - | - | - | - | - | - | - | |
| *Streptococcus constellatus* | Clinical isolate | - | - | - | - | - | - | - | - | - | - | - | - | - | - | - | - | - | - | |
| *Enterococcus faecium* | Clinical isolate | - | - | - | - | - | - | - | - | - | - | - | - | - | - | - | - | - | - | |
| *Streptococcus gallolyticus* | Clinical isolate | - | - | - | - | - | - | - | - | - | - | - | - | - | - | - | - | - | - | |
| *Staphylococcus saprophyticus* | Clinical isolate | - | - | - | - | - | - | - | - | - | - | - | - | - | - | - | - | - | - | |
| *Cupriavidus* | Clinical isolate | - | - | - | - | - | - | - | - | - | - | - | - | - | - | - | - | - | - | |
| *Listeria monocytogenes* | ATCC 19115 | - | - | - | - | - | - | - | - | - | - | - | - | - | - | - | - | - | - | |
| *Bacillus cereus* | ATCC 11778 | - | - | - | - | - | - | - | - | - | - | - | - | - | - | - | - | - | - | |
| *Salmonella Enteritidis* | ATCC 13076 | - | - | - | - | - | - | - | - | - | - | - | - | - | - | - | - | - | - | |
| *Cronobacter sakazakii* | ATCC 29544 | - | - | - | - | - | - | - | - | - | - | - | - | - | - | - | - | - | - | |
| *Staphylococcus aureus* | ATCC 29213 | - | - | - | - | - | - | - | - | - | - | - | - | - | - | - | - | - | - | |
| *Escherichia coli* O157:H7 | ATCC 700728 | - | - | - | - | - | - | - | - | - | - | - | - | - | - | - | - | - | - | |

**Table S4** Bacterial strains used for specificity tests of PCR and qPCR.


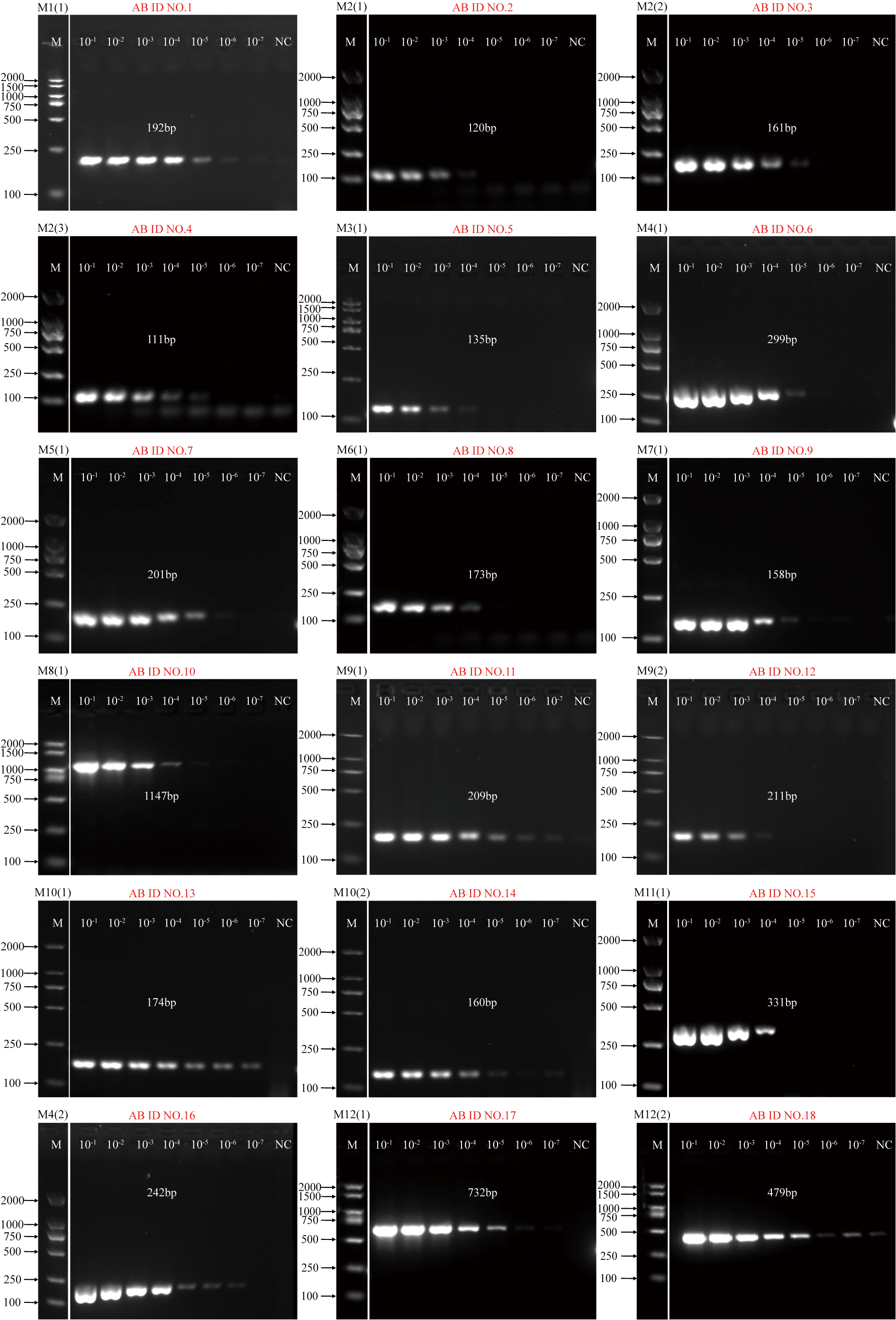

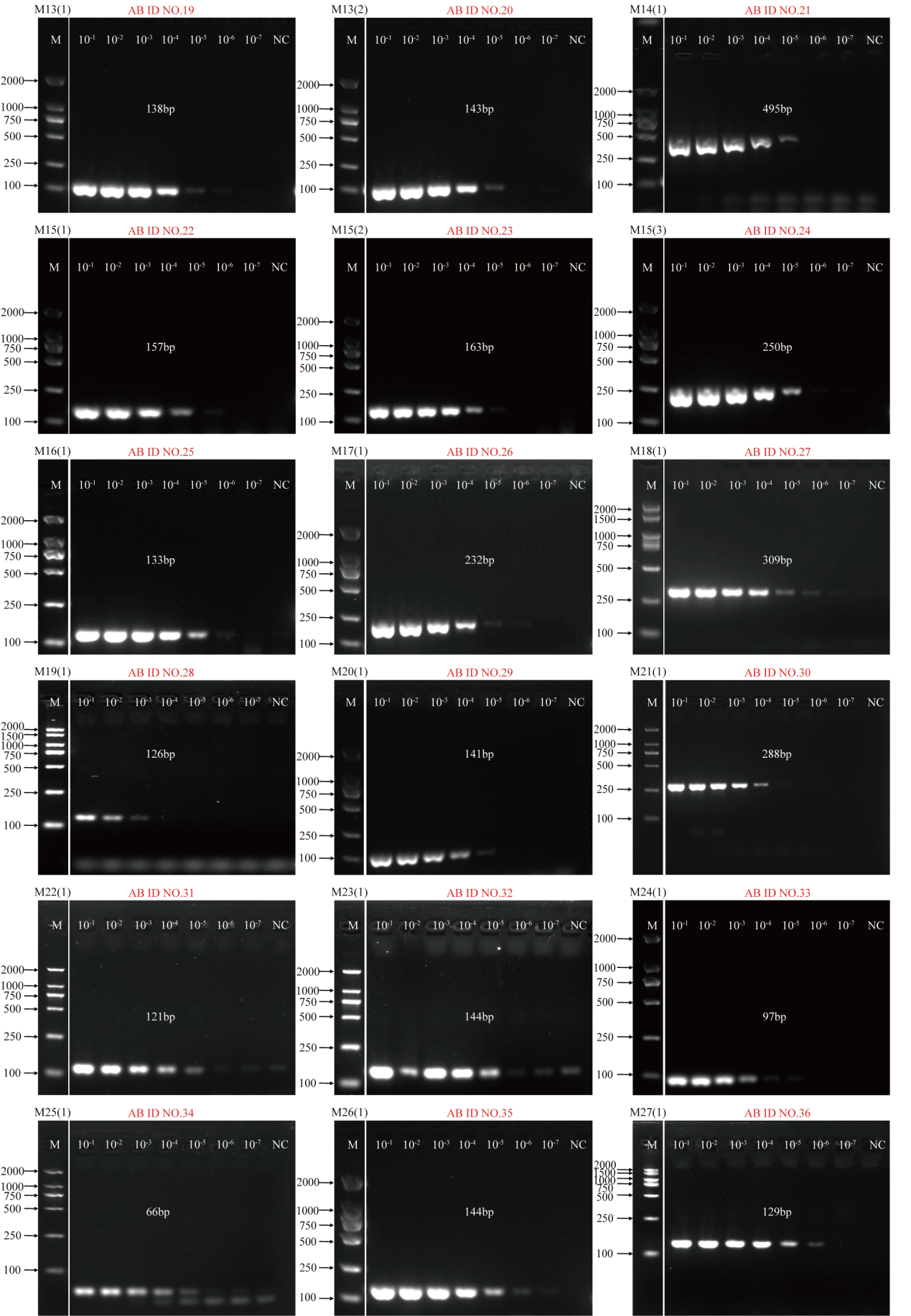


**Fig. S1** Electrophoresis results of the sensitivity detection of PCR primers and qPCR primers for *Acinetobacter baumannii*. Lane M: 100-2000 bp marker; lanes 1-7 respectively represent the sample serial number and NC represent the negative control.


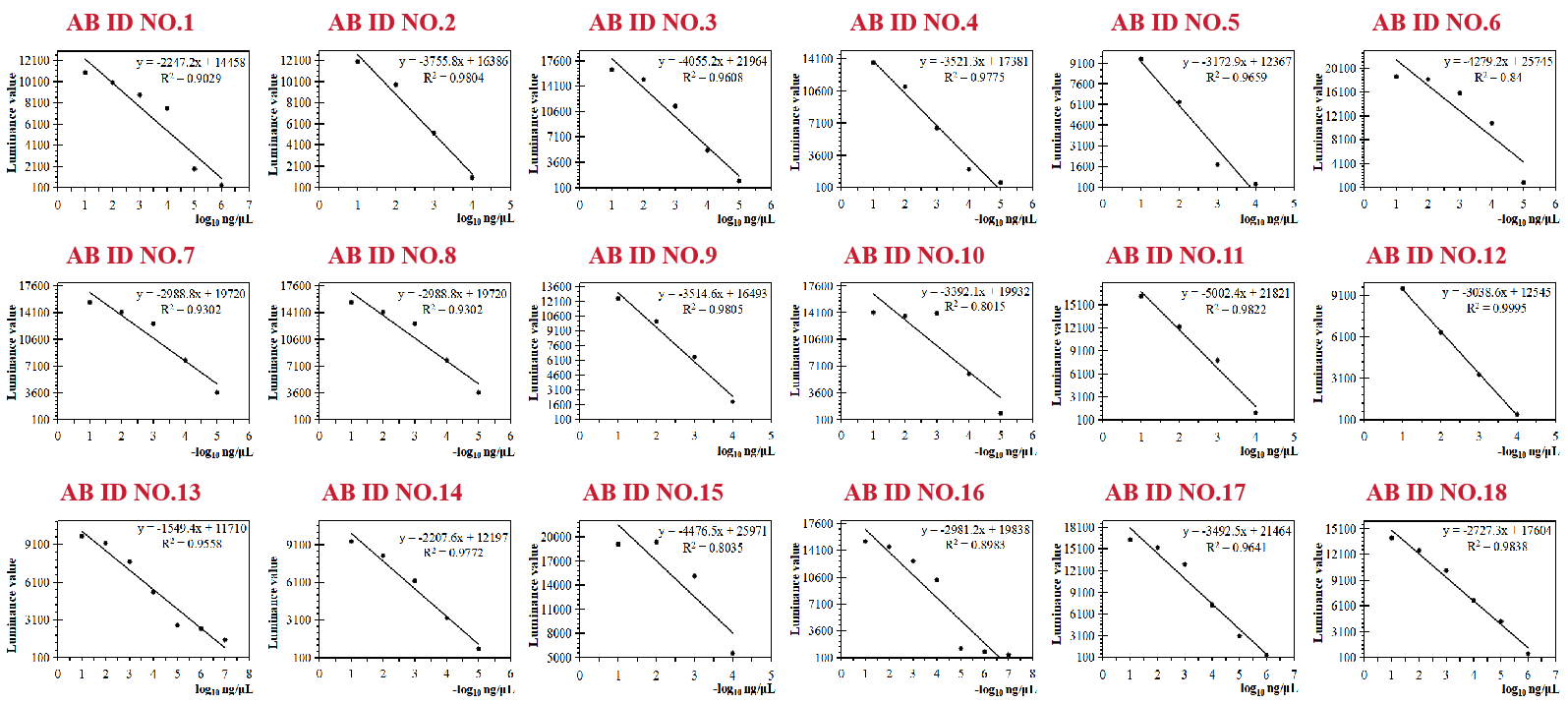

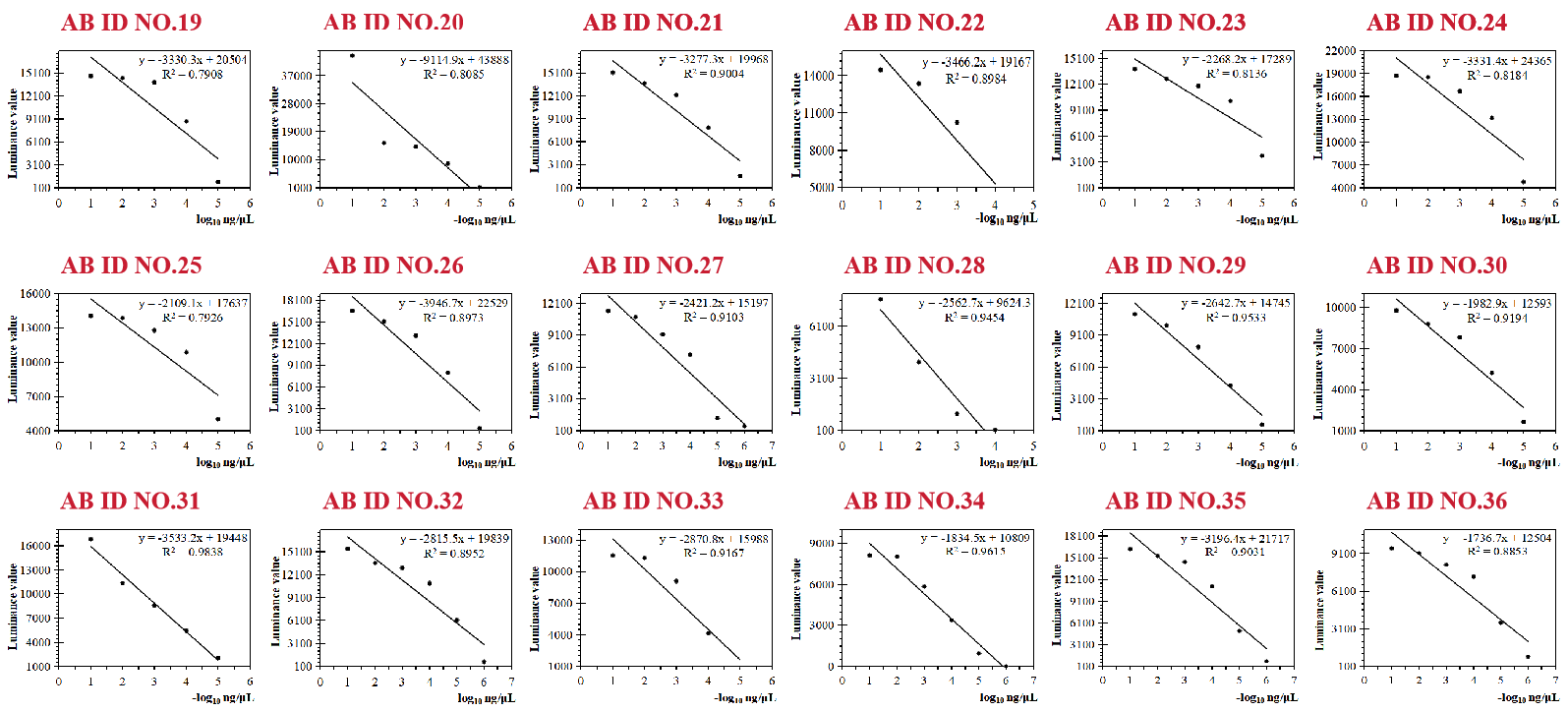


**Fig. S2** The standard curves for the sensitivity detection of the PCR primers and qPCR primers of *Acinetobacter baumannii*. AB ID NO.1~27 are PCR primers, AB ID NO.28~36 are qPCR primers.

**
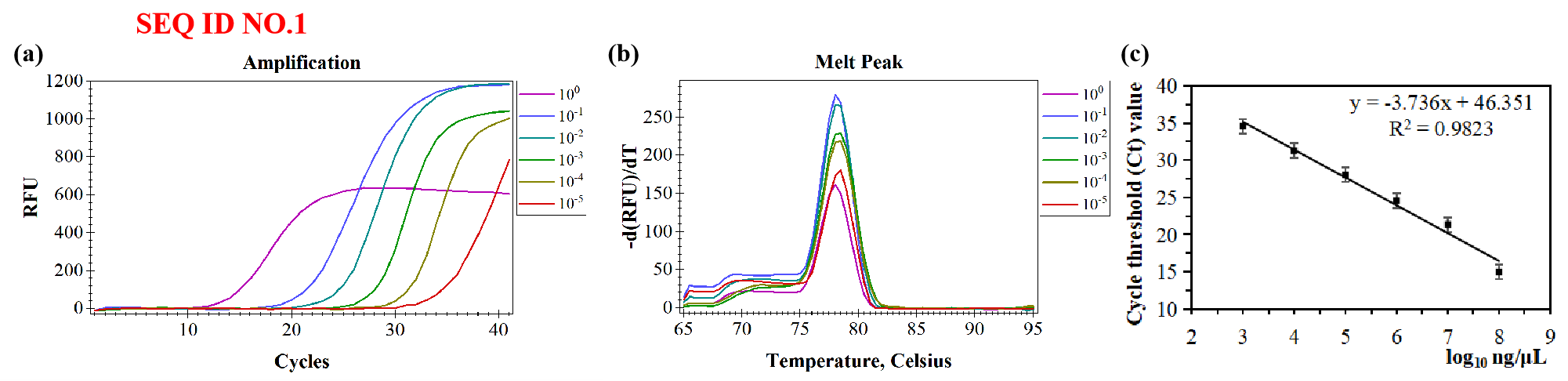

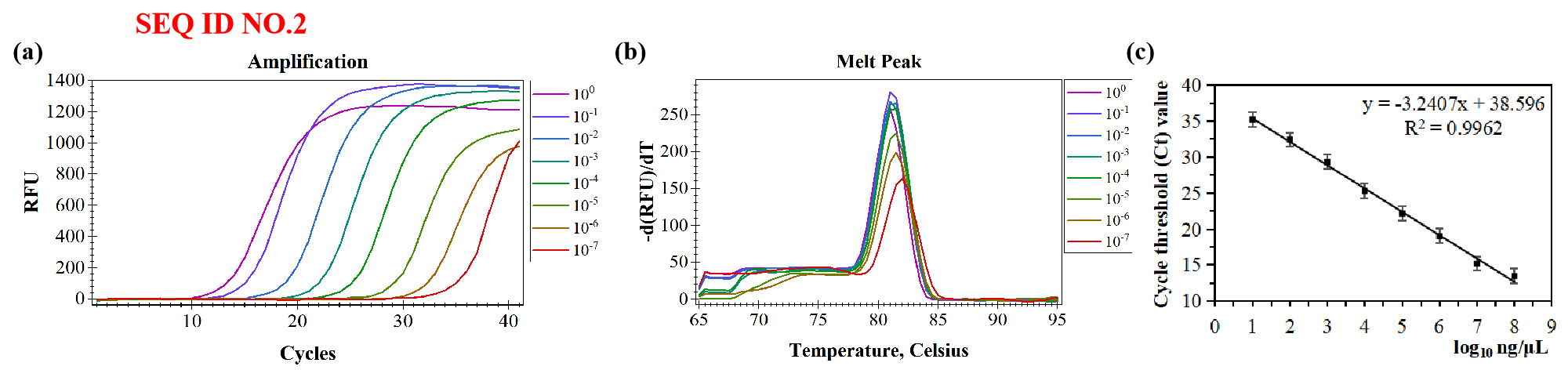

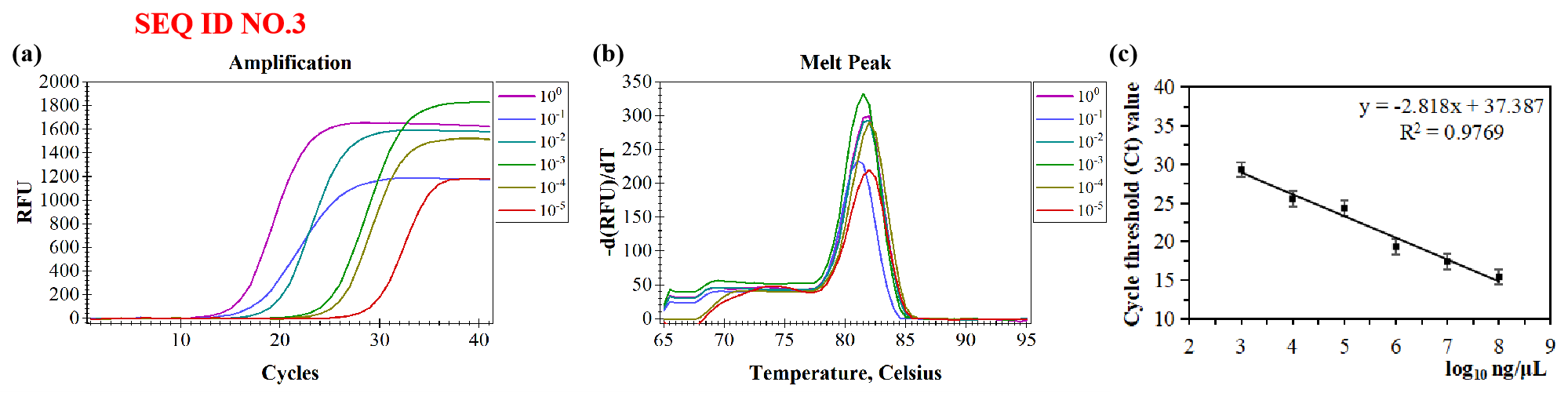

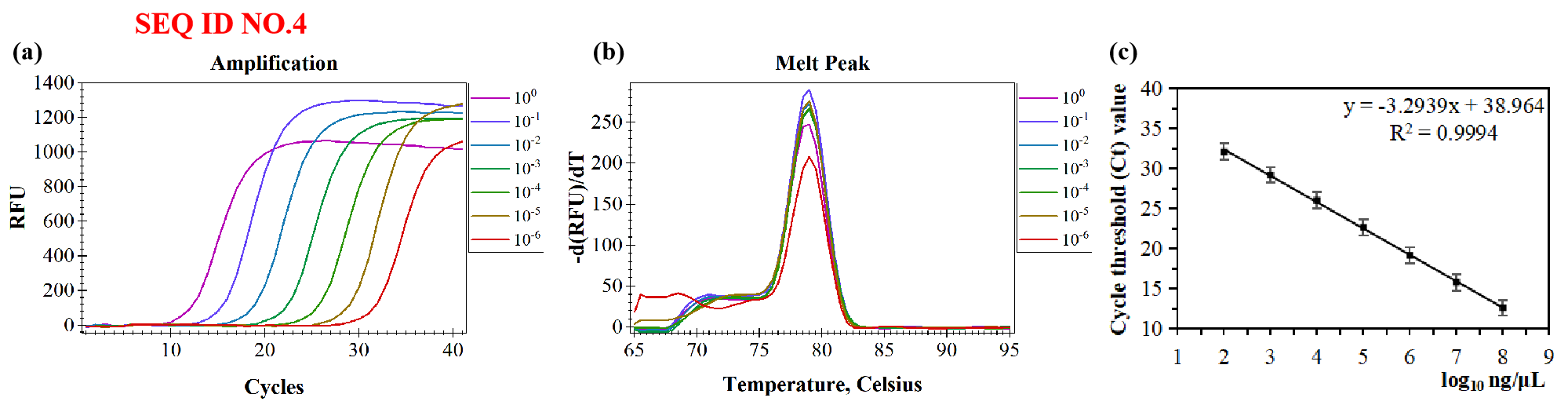

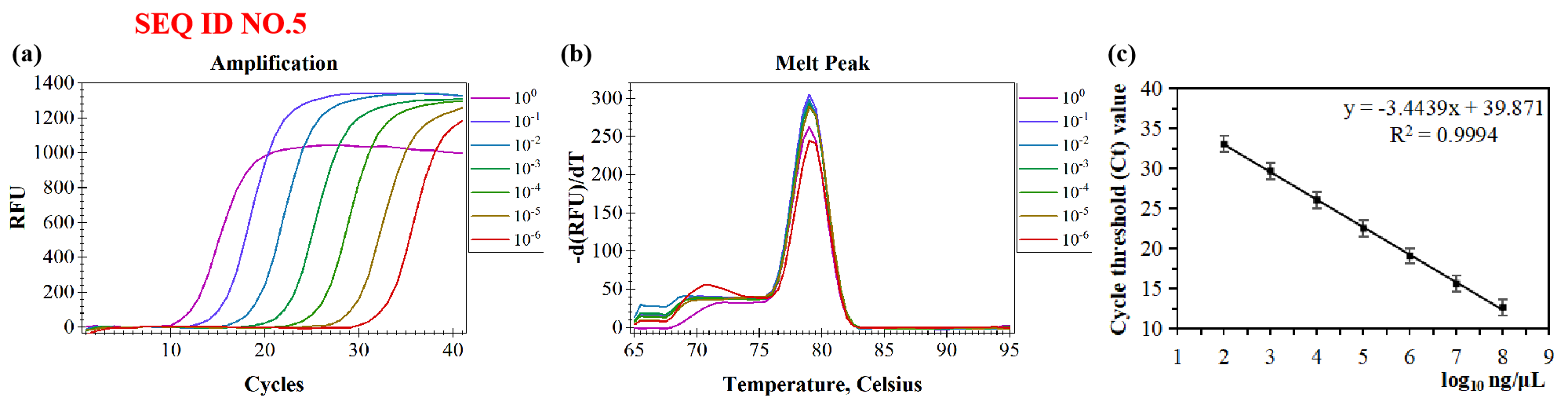

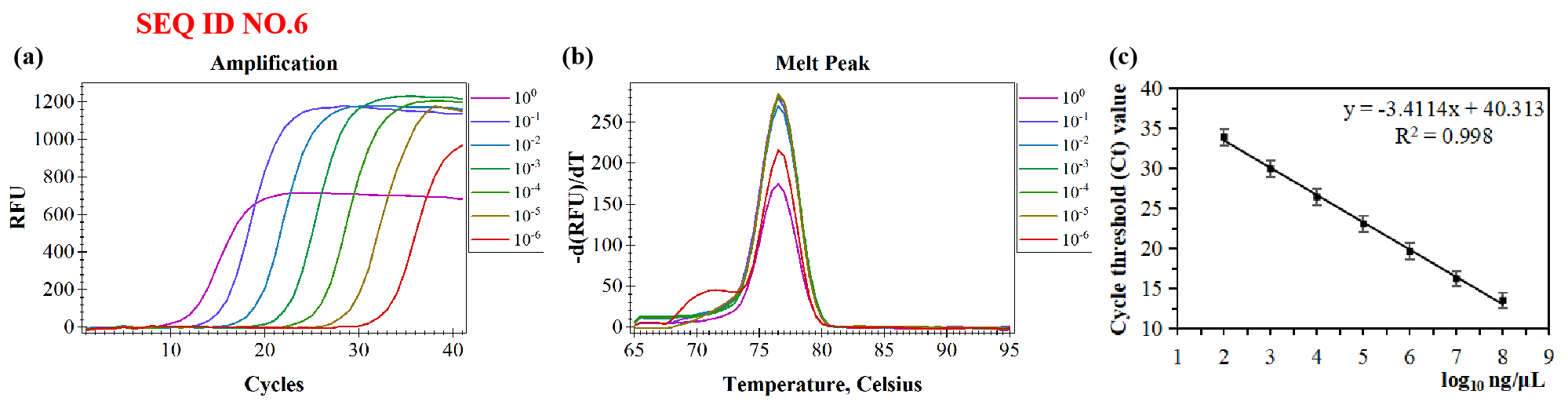

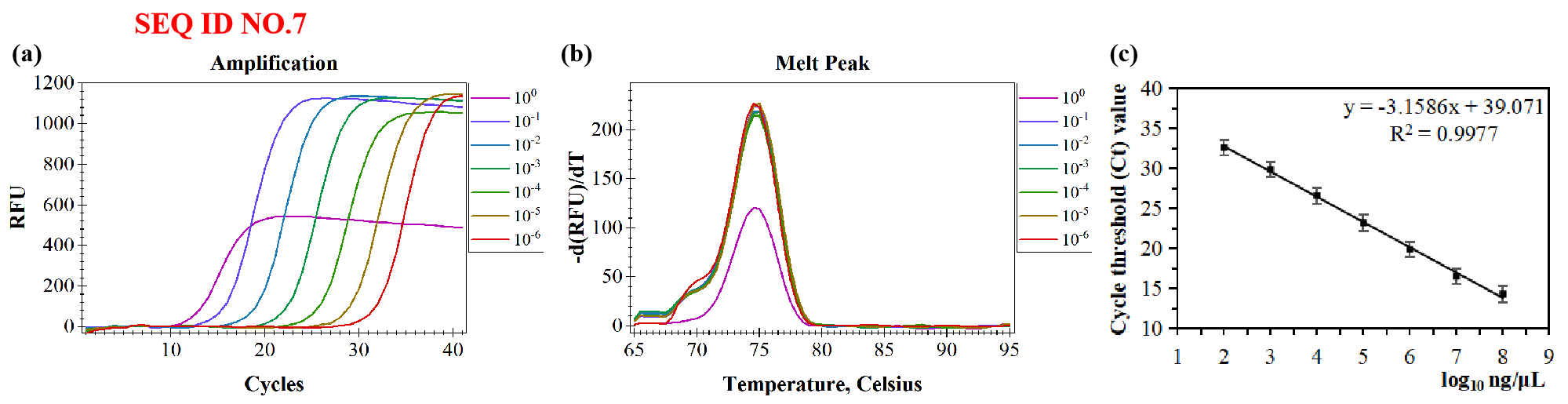

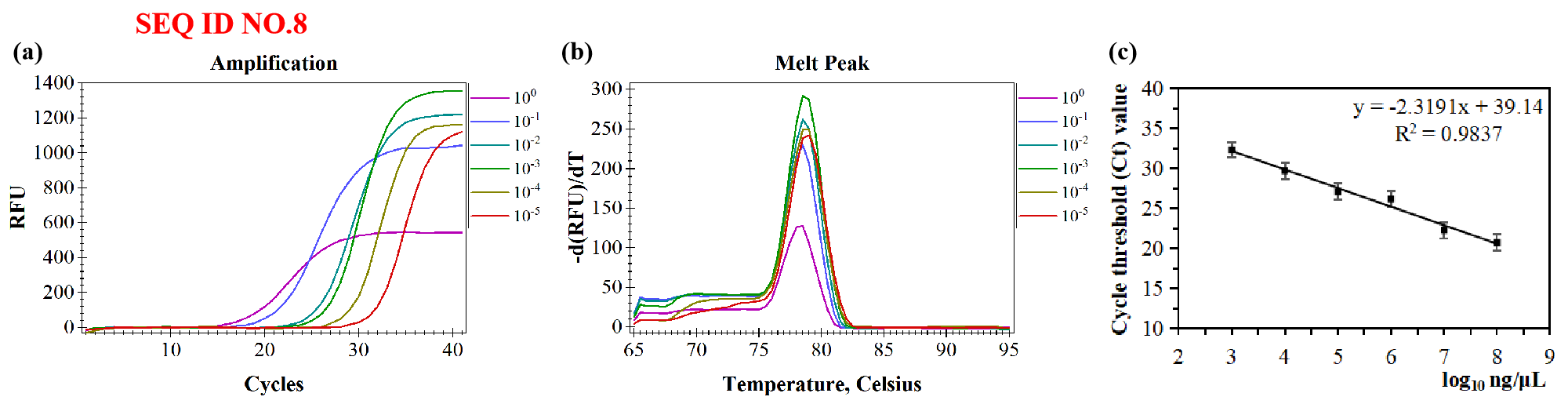

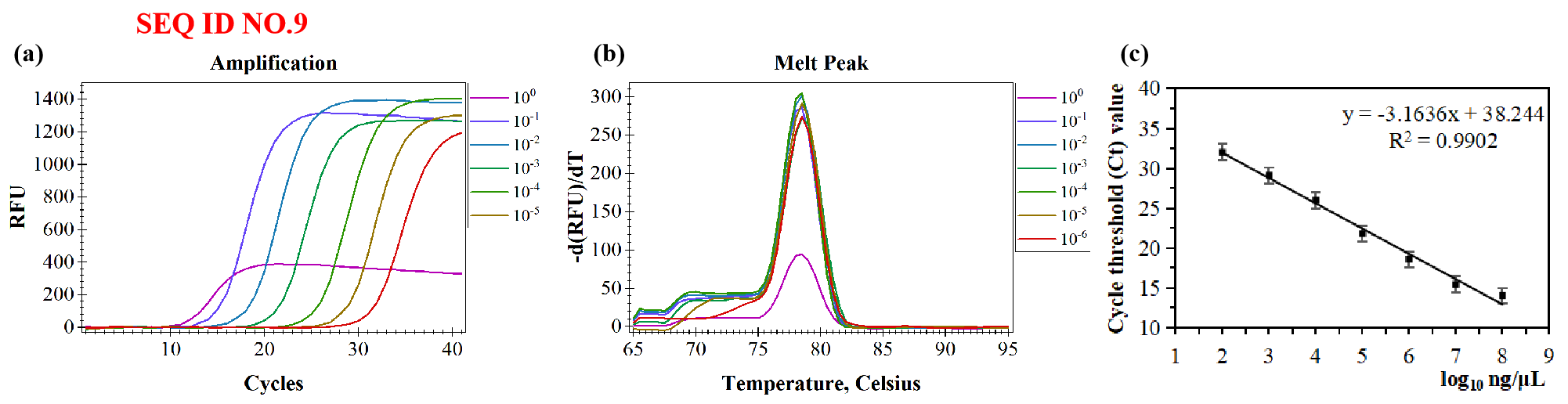
Fig. S3** Sensitivity of 9 pairs of optimal primers detected by qPCR method. SEQ ID NO.1~9 were screened from 3 pairs of PCR primers and 1 pair of qPCR primers designed based on the nine *Acinetobacter baumannii* target genes, which are outO, ureE, rplY, bioF, menH_3, hemW, paaF_1, smpB, and ppaX respectively. (a) Amplification curve of products; (b) Melting curve of products; (c) Standard curve plotted based on Ct value and log_10_ng/µL of qPCR amplification product.
